# Treatment for Anomia in Bilingual Speakers with Progressive Aphasia

**DOI:** 10.3390/brainsci11111371

**Published:** 2021-10-20

**Authors:** Stephanie M. Grasso, Elizabeth D. Peña, Nina Kazemi, Haideh Mirzapour, Rozen Neupane, Borna Bonakdarpour, Maria Luisa Gorno-Tempini, Maya L. Henry

**Affiliations:** 1 Department of Speech, Language and Hearing Sciences, University of Texas, Austin, TX 78705, USA; kazeminina@gmail.com (N.K.); haideh39@yahoo.com (H.M.); neupane.rozen@gmail.com (R.N.); maya.henry@austin.utexas.edu (M.L.H.); 2 School of Education, University of California, Irvine, CA 92697, USA; edpena@uci.edu; 3 Feinberg School of Medicine, Northwestern University, Chicago, IL 60611, USA; bbk@northwestern.edu; 4 Memory and Aging Center, Department of Neurology, University of California San Francisco, San Francisco, CA 94143, USA; MariaLuisa.GornoTempini@ucsf.edu; 5 Department of Neurology, Dell Medical School, University of Texas, Austin, TX 78705, USA

**Keywords:** bilingualism, primary progressive aphasia, treatment, intervention

## Abstract

Anomia is an early and prominent feature of primary progressive aphasia (PPA) and other neurodegenerative disorders. Research investigating treatment for lexical retrieval impairment in individuals with progressive anomia has focused primarily on monolingual speakers, and treatment in bilingual speakers is relatively unexplored. In this series of single-case experiments, 10 bilingual speakers with progressive anomia received lexical retrieval treatment designed to engage relatively spared cognitive-linguistic abilities and promote word retrieval. Treatment was administered in two phases, with one language targeted per phase. Cross-linguistic cognates (e.g., rose and *rosa*) were included as treatment targets to investigate their potential to facilitate cross-linguistic transfer. Performance on trained and untrained stimuli was evaluated before, during, and after each phase of treatment, and at 3, 6, and 12 months post-treatment. Participants demonstrated a significant treatment effect in each of their treated languages, with maintenance up to one year post-treatment for the majority of participants. Most participants showed a significant cross-linguistic transfer effect for trained cognates in both the dominant and nondominant language, with fewer than half of participants showing a significant translation effect for noncognates. A gradual diminution of translation and generalization effects was observed during the follow-up period. Findings support the implementation of dual-language intervention approaches for bilingual speakers with progressive anomia, irrespective of language dominance.

## 1. Introduction

The majority of individuals worldwide speak two or more languages (e.g., [1,2]); nonetheless, most studies that have evaluated the benefits of speech-language intervention for individuals with aphasia have focused on monolingual speakers (e.g., [3,4,5,6,7,8]). This disparity is even more striking in aphasia caused by neurodegenerative disease (e.g., [9,10]). In the United States, bilingual speakers are more likely to belong to historically minoritized populations (e.g., [10]). Therefore, the lack of evidence regarding treatment for bilingual speakers with aphasia disproportionately impacts individuals from historically marginalized populations, which, in turn, contributes to health disparities in these groups. In an era of globalization, speech-language pathologists are increasingly called upon to provide services for individuals who speak more than one language [11,12]. This necessitates careful consideration of therapeutic manipulations that may be used to support multiple languages for bilingual speakers, especially given the shortage of bilingual speech-language pathologists in the United States [13].

In this study, we sought to investigate whether a lexical retrieval intervention that has largely been evaluated in monolingual speakers [14,15,16] would be efficacious for bilingual speakers with progressive anomia. The treatment was adapted to include distinct targets treated in each of the participants’ languages. We also examined whether inclusion of targets with shared phonology (i.e., cross-linguistic cognates, such as dentist and its Spanish translation equivalent *dentista*) may promote naming accuracy across languages. In the following sections, we briefly review neurodegenerative syndromes that may present with progressive anomia, summarize the literature examining restitutive interventions in monolingual and bilingual speakers with progressive anomia, and present evidence for treatment-induced cross-linguistic transfer in bilingual aphasia.

### 1.1. Progressive Anomia

Anomia is a ubiquitous feature of aphasia syndromes and distinct etiologies can result in word-retrieval difficulty. This study includes patients with anomia in the context of a number of neurodegenerative disorders. Primary progressive aphasia (PPA) is a neurodegenerative syndrome characterized by gradual worsening of speech and language ability, with relative sparing of other cognitive domains [17]. International consensus criteria delineate three clinical variants of PPA [18]: the nonfluent/agrammatic variant, the semantic variant, and the logopenic variant. Each subtype presents with a distinct profile of speech and/or language impairments and pattern of brain atrophy (e.g., [19]). Anomia is a core feature of both the logopenic and semantic PPA variants but for different underlying reasons. The logopenic variant of PPA (lvPPA) presents with a core deficit in phonological processing, which manifests as impaired word retrieval in spontaneous speech and naming, and impaired repetition of phrases and sentences [20]. In this syndrome, cortical atrophy is typically observed in left temporoparietal regions implicated in phonological processing and phonological working memory [20,21]. LvPPA is most often associated with Alzheimer’s pathology [22].

The semantic variant of PPA (svPPA) presents with left greater than right atrophy in the anterior temporal lobes [23,24]. Individuals with svPPA have impaired confrontation naming and single-word comprehension due to a gradual degradation of conceptual knowledge [18]. In cases where right anterior temporal atrophy is greater than that in the left hemisphere, individuals are characterized using different diagnostic terminology, either behavioral variant frontotemporal dementia or right temporal variant of FTD (e.g., [25,26]). These individuals are also anomic; however, their anomia is typically less pronounced than deficits in affect processing, and person and social semantic knowledge [25,26,27,28]. Both left and right temporal variants of FTD that present with primary deficits in semantic processing are associated with TDP-43 proteinopathy [22].

### 1.2. Treatment for Progressive Anomia in Primary Progressive Aphasia

At present, there are no pharmacological interventions proven to successfully treat the speech and language symptoms that accompany PPA or FTD. There is, however, a growing body of evidence documenting the utility of behavioral speech-language interventions to improve targeted communication skills in PPA. Most of this work has centered on treating anomia in the context of PPA, with the overwhelming majority of studies focusing on monolingual speakers (for reviews, see [29,30,31,32,33,34,35,36]).

Treatment for anomia has been shown to result in improved naming in all three PPA variants; however, given the scope of the current paper, we will focus on outcomes reported in sv and lvPPA. The treatment approaches used to treat anomia in sv and lvPPA range from rehearsal of spoken and/or written word forms [31,37,38,39,40,41,42,43,44,45,46,47,48,49] to more varied training tasks, some of which are designed to encourage self-cueing through the recruitment of residual semantic and word form knowledge [14,15,40,50,51,52]. Maintenance of treatment gains has been more frequently observed in lv versus svPPA, with gains observed up to 12 [15,53] and 15 months post-treatment [54], respectively. In both variants, generalization to untrained items has been reported. Generalization is more often reported in studies that have utilized approaches that incorporate more elaborated training tasks and/or that encourage self-cueing, e.g., [14,15,16,40,50,51,52,55].

Only one study [10] has examined the effects of naming intervention administered to a bilingual speaker (Norwegian-English) with PPA (i.e., lvPPA). The treatment, administered only in English, began with eight in-person sessions, which were then followed by 11 months of home practice. In general, the participant showed a decline in both languages from pre- to post-treatment, with the exception of written naming accuracy. More specifically, the participant demonstrated better written naming for trained versus untrained items in English. Despite the fact that treatment was only offered in English, evidence for cross-language transfer was observed in oral naming and naming-to-definition in Norwegian. The results of this study suggest that cross-language transfer is possible in bilingual PPA, despite progressive worsening. 

In sum, research addressing speech-language treatment for monolingual speakers with PPA documents that intervention is efficacious and may have long-term benefits for some individuals. In bilingual speakers, additional research is needed in order to evaluate the effects of intervention within and between languages, and to investigate optimal treatment designs to promote cross-linguistic transfer.

### 1.3. Cross-Linguistic Transfer in Treatment for Anomia in Bilingual Aphasia and the Role of Cognates

Studies of linguistic processing in healthy bilingual speakers can inform predictions regarding treatment-induced cross-linguistic transfer in bilingual aphasia. Perhaps the closest analogue to cross-linguistic transfer in neurotypical bilingual speakers is that of translation. Evidence from studies of healthy bilingual speakers has shown asymmetry in translation directionality, such that backward translation (L2 to L1) is faster and more accurate than forward translation (L1 to L2, e.g., [56,57,58,59]), particularly for those who learn their L2 subsequent to their L1. This pattern is thought to reflect weakened links between the L2 lexicon and conceptual representations relative to the L1. This is in addition to stronger lexical links from the L2 to the L1 (bilingual speakers may access conceptual information via the L1, particularly at lower levels of L2 proficiency, as is described in the revised hierarchical model [58]). Interestingly, bilingual speakers who speak languages that share cross-linguistic cognates (i.e., words that share meaning and form across languages, such as telephone and *teléfono*) tend to demonstrate a cognate facilitation effect, wherein cognates are named faster and are translated more quickly and reliably relative to noncognates (e.g., [56,57,60,61,62,63,64,65,66,67]). This may be possible due to shared conceptual representations activating lexical items in both languages, with cognates benefiting from increased activation from shared phonological segments.

Studies examining cross-linguistic transfer effects following treatment for anomia in stroke-induced aphasia have reported different patterns of transfer (e.g., [3,4,5,68]). The majority of naming intervention studies report transfer or generalization from participants’ trained L2 to their untrained L1 (e.g., [69,70,71,72,73]). Other studies have found transfer to the untrained L2 following L1 treatment (e.g., [74,75,76]). Taken together, these studies illustrate that bidirectional transfer is possible, but not uniform, in the context of aphasia treatment. In addition, a series of studies (e.g., [72,75,77]) has shown that the effects of an intervention targeting semantic bases of naming can result in within- and between-language transfer (to translation equivalents of trained items and to untrained items). Other work has investigated whether the inclusion of cognates in treatment may result in greater cross-linguistic transfer effects.

The effect of including cognates as treatment targets for bilingual speakers with aphasia has been examined primarily in the context of naming intervention. A handful of studies has reported positive transfer effects for cognate items [78,79] in individuals with nonfluent aphasia. Other studies have not observed such an effect [69,80,81]. The variability of cognate transfer effects reported from single cases in the literature may be attributed to a number of factors, including participant characteristics and differences in methodology (e.g., treatment approaches/tasks). Given that the majority of studies examining treatment for bilingual aphasia have focused on stroke-induced aphasia, a study examining the effect of treatment and potential for cross-language transfer in progressive aphasia is warranted.

### 1.4. The Present Study

The aim of this study was to investigate the effects of an established lexical retrieval training approach in a series of bilingual speakers with progressive anomia. Each individual underwent treatment using a single-subject multiple baseline design, with treatment administered in each of their languages in distinct phases. We assessed performance on items trained in each language, as well as cross-linguistic transfer effects (performance of untrained translation equivalents in one language that were trained items in the other language). Performance on trained and untrained items as well as standardized tests was assessed before, during, and after treatment, with follow-up testing at 3, 6, and 12 months post-treatment. Consistent with previous research [15], we predicted that treatment would result in improved naming for trained items, with maintenance of gains in the follow-up period, and with some participants demonstrating evidence of generalization to untrained items. We also hypothesized that treated cognates would show significant cross-linguistic transfer and that the magnitude of transfer would be significantly greater than that for noncognates.

## 2. Materials and Methods

### 2.1. Participants

Ten bilingual speakers with progressive anomia were recruited for this study. Participants included one individual with the right temporal variant of frontotemporal dementia, four participants with the semantic variant of PPA, and five participants with the logopenic variant PPA. With the exception of the participant with right temporal variant FTD, participants with PPA met current diagnostic criteria for PPA and subtype [17,18]. Inclusionary criteria required that individuals presented with progressive anomia, and attained a conceptual or composite score ([82,83,84]; where an appropriate response in either language is counted as correct) of 15 or higher on the Mini-Mental State Exam [85] at pre-treatment. In addition, we recruited only bilingual individuals who reported speaking both languages at the time of enrollment and who were in favor of undergoing treatment in both of their languages. Bilingual individuals who reported no longer using one of their languages were not enrolled in the current study but were enrolled in a separate study evaluating the effects of intervention provided in English only.

**Figure 1:**
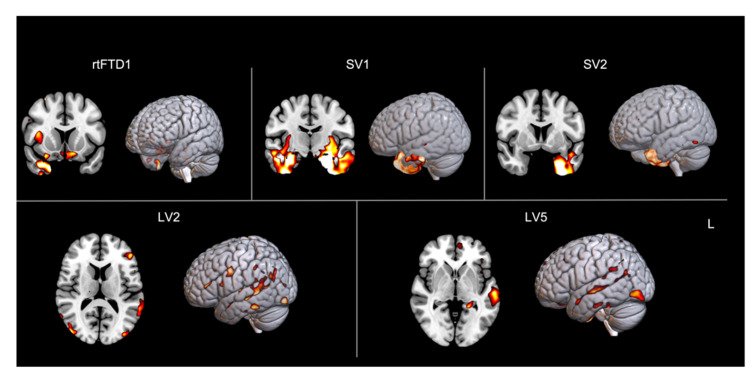
Results of whole brain voxel-based morphometry analysis showing atrophy patterns for each participant relative to controls (*n* = 30, FWE < 0.05, k = 100, total intracranial volume and age included as covariates). Note that scans were available for only five participants. rtFTD = right temporal variant of frontotemporal dementia; SV = semantic variant; LV = logopenic variant.

Six individuals were male and nine were right-handed, with one participant reporting ambidexterity. The mean age of participants was 67 years (±7) and, on average, individuals were 3.5 years (±2) post symptom onset. All participants spoke English and another language (*n* = 5 Spanish, *n* = 2 Farsi, *n* = 1 Portuguese, and *n* = 1 French; see Table 1). Participants gave written informed consent, and all procedures were approved by the institutional review board at The University of Texas at Austin. Structural magnetic resonance imaging was acquired for five participants prior to the commencement of treatment and voxel-based morphometry analysis was conducted, comparing each participant to 30 healthy age-matched controls (see Figure 1). The results from these analyses revealed the expected pattern of atrophy for each individual (left > right anterior temporal lobe atrophy in svPPA (right > left for right temporal variant) and left > right temporoparietal atrophy in lvPPA). All participants lived at a distance from the research site; therefore, assessment and treatment were conducted via HIPAA-compliant videoconferencing software (Fuze, Adobe Connect or Zoom). Previous work from our group has shown that treatment delivery modality (face-to-face versus telerehabilitation) does not impact treatment outcomes (i.e., performance on the primary outcome measure and maintenance and generalization effects) for the intervention used in this study [86].

A language use history questionnaire (subset of items from Kiran et al. [87]) was used to gain information regarding individuals’ use and exposure to each of their languages. A summary of each individual’s language history is provided in Table 1. There was a range in age of second language acquisition (birth-18 years) and seven participants reported dominance in English. All participants received a comprehensive cognitive-linguistic evaluation prior to the initiation of treatment in order to confirm diagnosis and clinical subtype. Aphasia with prominent anomia and a history of progressive decline were confirmed in all participants. In general, participants demonstrated better performance in their dominant language. Pre-treatment assessment scores are presented in Table 2.

### 2.2. Treatment Design and Procedures

**Figure 2:**
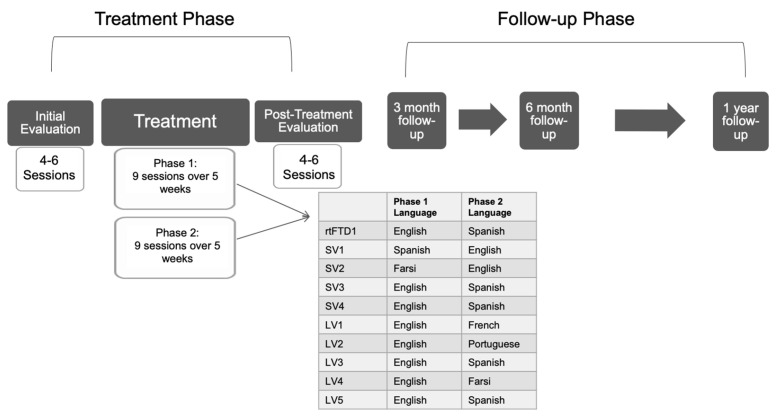
Schematic depicting chronology and duration of participation. rtFTD = right temporal variant of frontotemporal dementia; SV = semantic variant; LV = logopenic variant.

Treatment was administered following a single subject multiple-baseline design, with two intervention phases (one language per phase; see Figure 2 for the training schedule). An adapted form of Lexical Retrieval Cascade Treatment [14,15] was used to target individually tailored word sets for all participants. The treatment cascade targets naming via guided retrieval of residual semantic, phonological, and orthographic information, with the goal of retraining specific vocabulary as well as instilling strategies for word retrieval more broadly (see Table 3 for the sequence of training tasks). Treatment sessions occurred twice weekly. Daily homework consisted of Copy and Recall Treatment [88], involving repeated rehearsal and delayed recall of spoken and written target words.

Treatment targets consisted of six sets of words, each containing 4 or 8 nouns (participants had different numbers of words per set for pragmatic reasons related to severity of anomia and the number of viable cognates that existed across different language pairs); therefore, the total treatment set contained either 24 or 48 nouns. Untrained items for each participant comprised a minimum of two sets (again containing 4 or 8 nouns); therefore, the total untrained set contained 8 to 24 items. Participants and their care partners provided images of items for inclusion in treatment; when possible, these items were prioritized for inclusion and were distributed across trained and untrained sets. When an insufficient number of items from the personal set were provided, functional items were supplemented by the clinician. In general, items were eligible for inclusion in treatment if participants did not name the item on two out of three occasions in both languages. However, for the first two participants, we required that they not name the item on two out of three occasions in the target language only (i.e., the language the item was assigned to for training; rtFTD1 and SV1). This means that some items treated in Spanish were accurately named in English on two out of three attempts and vice versa. For these two individuals, only the consistently unnamed subset was included when examining cross-language translation effects. As a result, for SV1, an insufficient number of items was present to assess translation effects for noncognates from Spanish to English.

For each language of treatment, half of the treated and untreated items were cross-linguistic cognates. Sets were trained for three sessions each in their assigned language. All word sets (trained and untrained) were balanced for frequency, length in letters (English, French, Spanish, and Portuguese), or phonemes (English and Farsi) within and across languages. When possible (i.e., when corpora contained these variables), sets were also balanced within and across languages for familiarity, imageability, and concreteness (English, Spanish, and French). Psycholinguistic parameters were attained from the following sources in each language: English = Medical Research Council Psycholinguistic Database [89], Corpus of Contemporary American English [90], and the CLEARPOND database [91]; Spanish= Corpus del Español [92], the CLEARPOND database, and EsPal [93]; French= Lexique [94], and the CLEARPOND database; Portuguese = Corpus do Portugues [95], and Farsi= TalkBank Persian [96,97].

The lead author (S.G.) administered treatment in both phases for individuals who spoke English and Spanish, and in the English phase for individuals who spoke English and a different language. For those participants who spoke English and a different language (French, Portuguese, and Farsi), clinicians were recruited and trained to administer treatment in the non-English treatment phase; in one case, a doctoral student in French linguistics assisted with assessment and treatment after extensive training and observation.

After the formal treatment period ended, participants were allowed to retain their homework materials and to practice their trained items. Allowing practice to take place after the immediate treatment period likely mirrors what occurs in typical clinical care with speech-language pathologists, wherein individuals are allowed and encouraged to practice with their treatment materials. This was consistent with procedures from the original studies demonstrating efficacy for this treatment approach [14,15].

### 2.3. Treatment Fidelity

Undergraduate and graduate students in speech-language pathology or linguistics, who spoke the language of treatment administration, were trained to conduct treatment fidelity ratings. Raters were provided with a template that included each treatment step (in the prescribed order). While reviewing each video, the rater indicated whether the clinician performed each step. If the same clinician provided treatment to a participant in both phases of treatment, then 25% (5/18) of the total number of sessions (sampled across phases) were independently reviewed by one student. If different clinicians administered each phase of treatment, 33% (3/9) of the total number of sessions were reviewed from each *phase* of treatment, except for two participants. For these two participants, videos were only available for 11% (1/9) or 22% (2/9) of sessions from one phase of treatment; however, a full set of videos was available for the other phase of treatment. The percentage of correctly administered treatment steps was calculated for each reviewed session. Fidelity ratings, averaged across participants, revealed that clinicians adhered to the treatment steps with 99.21% accuracy.

### 2.4. Self- and Communication Partner-Assessment of Change Following Treatment

Participants and their primary communication partners were asked to complete a post-treatment survey [14,15] documenting their perceptions regarding changes in communication from pre- to post-treatment. The survey consisted of 20 questions and a qualitative rating scale was used to capture respondents’ perceptions (7 point scale: 3 = “A lot better,” 2 = “Better,” 1 = “Somewhat better,” 0 = “Unchanged,” −1 = “Somewhat worse,” −2 = “Worse,” and −3 = “A lot worse”).

### 2.5. Follow-Up Assessment

Follow-up assessments were conducted at 3, 6, and 12 months post-treatment. Only one participant (lv1) was unavailable for follow-up assessment at 3 and 6 months post-treatment, due to health-related issues. Additionally, one individual had yet to complete the follow-up period (sv5) at the time that this paper was written. All remaining participants were available at one year post-treatment. Performance on standardized assessments at each time point is reported in Appendix A.

### 2.6. Outcome Measures and Statistical Analysis

Data were analyzed at the single-subject level. The primary outcome measure was the proportion of items named correctly during probing for trained and untrained stimuli in the target language. Cross-linguistic transfer was assessed by examining participants’ responses to treatment probes for trained stimuli in the non-targeted language (i.e., translation effects). Probes were collected in each language three times at pre-treatment, once or twice at mid-treatment, twice at post-treatment, and once at each follow-up visit (3, 6, and 12 months post-treatment). Additionally, approximately half of items were probed at the beginning of each treatment session in a given language, so that all sets were probed once per week in each language.

Significance testing was conducted using a simulation technique [98]. An individual’s percent accuracy was attained from each condition and probabilities of correct responses were used to create simulated datasets with parameters that mirror the observed data. This procedure was completed 10,000 times to create 10,000 simulated distributions of accuracy scores from each condition, at each time point. The resulting simulated datasets from two conditions were then directly compared to one another to calculate a *p*-value (i.e., the likelihood that post-treatment performance was greater than pre-treatment performance). In addition, using the simulated data, difference scores were calculated between conditions to determine the 95% confidence intervals of the observed differences. For comparing differences in the magnitude of effects (e.g., translation effects between cognate and noncognate items), the same process was followed with one additional step. Specifically, simulations were conducted for each condition and time point, but *p*-values were calculated by comparing difference scores between time points and conditions (e.g., the difference scores for trained and untrained stimuli for simulated post-treatment minus simulated pre-treatment performance).

We predicted that each participant would demonstrate a significant treatment effect, with maintenance in the follow-up period. We also predicted that some participants would demonstrate evidence of generalization to untrained items. It was hypothesized that each participant would show a significant cross-language translation effect for cognate items and that the magnitude of this effect would be greater for cognates relative to noncognates.

In addition, we assessed performance over time (pre-treatment versus subsequent time points) on a subset of assessments administered in the dominant and nondominant language using paired permutation tests at the group level in order to identify overall trends with respect to stability and/or progression. Specifically, the stability of general cognitive and linguistic function (MMSE and WAB-R; [85,99]) and overall naming ability (Boston Naming Test; BNT [100]) were evaluated. We predicted that performance on the BNT would improve at post-treatment, consistent with previous literature demonstrating generalization on this measure in monolingual speakers with PPA [15]. Analyses comparing subsequent timepoints on the BNT and for all timepoints for the other assessments (MMSE and WAB-R) were assessed using two-tailed tests, as performance on these measures was less predictable over time.

## 3. Results

In the following sections, outcomes that are directly related to the aforementioned hypotheses will be reported. In order to contextualize our reporting of the number of participants demonstrating significant improvement at the individual level, we also provide the average change and range of performance for the entire group. For additional treatment outcomes (including outcomes following the first treatment phase and cross-linguistic generalization effects to untrained items), please see the Supplementary Materials.

### 3.1. Treatment and Maintenance Effects

**Figure 3:**
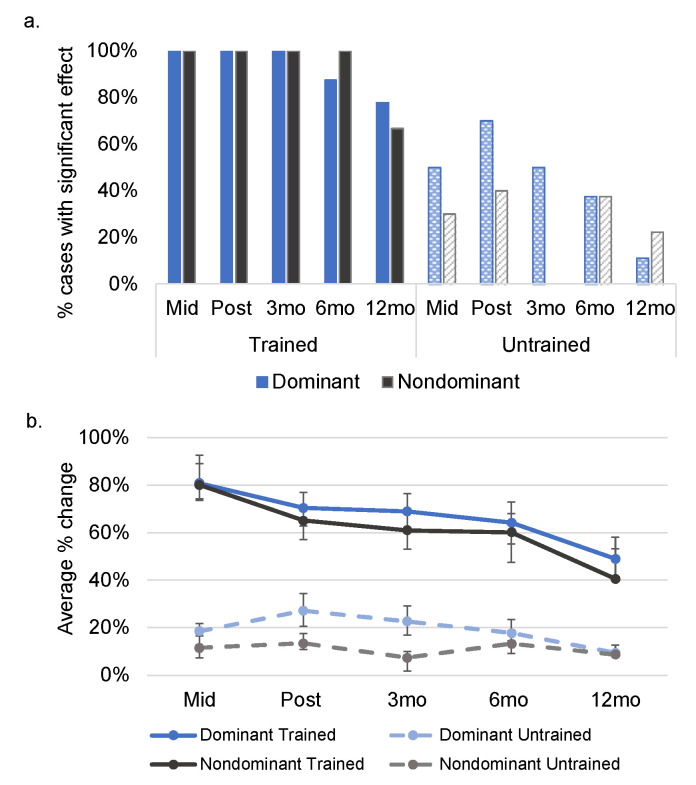
Within-language treatment and generalization effects at each time point relative to pre-treatment. (**a**). Depicts the percentage of cases demonstrating a significant effect at each time point relative to pre-treatment. (**b**). Depicts the average percent change at each time point relative to pre-treatment. At mid-treatment, seven of nine participants had received treatment in their dominant language and three had received treatment in their nondominant language; the figure shows performance for these subsets at mid-tx for trained items. See Supplemental Materials for data at the single-subject level. Mid = mid-treatment; mo = month.

Simulation analyses revealed that each participant demonstrated a significant treatment response in both their dominant (*M* change = 70.37%; range = 31–92%) and nondominant language (*M* change = 65.03%; range = 30–97%, see Figure 3) from pre- to post-treatment (after training in both languages was completed). Of the eight participants for whom follow-up data were collected at 3 and 6 months post-treatment, all participants had significantly better performance at the 3-month follow-up, and all but one individual had significantly better performance at the 6-month follow-up relative to pre-treatment. At 12 months post-treatment, seven of nine participants demonstrated significantly better performance relative to pre-treatment in their dominant language, with six of eight participants showing this pattern in their nondominant language.

### 3.2. Within-Language Generalization to Untrained Items

Seven of 10 individuals showed improvement on matched, untrained items in their dominant language (*M* change = 27.18%, range = −3–75%; see Figure 3), with four individuals showing this pattern in their nondominant language (*M* change = 13.50%, range = −4–38%), from pre- to post-treatment. A direct comparison of the magnitude of improvement on trained versus untrained items revealed a significant difference (with greater improvement for trained items) for six individuals from pre- to post-treatment in the dominant language (an additional three participants demonstrated a marginal or trending difference between trained and untrained items; *M* difference = 43.19%, range = –0.1–89%), and eight participants showing this pattern in the nondominant language (the remaining two participants demonstrated a marginal difference between trained and untrained items; *M* difference = 51.53%, range = 27–87%). Performance on untrained items showed gradual decline in the follow-up period.

### 3.3. Cross-Linguistic Translation Effects

**Figure 4:**
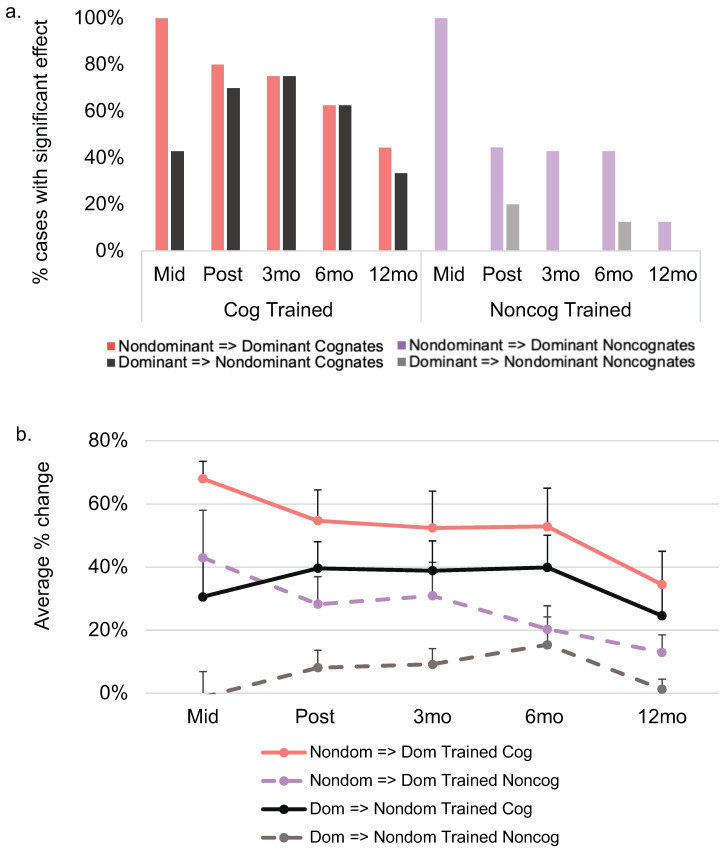
Cross-linguistic transfer effects by cognate status at each time point relative to pre-treatment. (**a**). Depicts the percentage of cases demonstrating a significant effect at each time point relative to pre-treatment. (**b**). Depicts the average percent change at each time point relative to pre-treatment. Performance on trained items across languages represents translation effects. At mid-treatment, seven of nine participants had received treatment in their dominant language and three had received treatment in their nondominant language; the figure shows performance for these subsets at mid-tx for trained and untrained items. See Supplementary Materials for data at the single-subject level. Mid = mid-treatment; mo = month.

Following both treatment phases, eight of 10 participants demonstrated a significant cross-linguistic translation effect relative to pre-treatment for cognates from the nondominant to the dominant language (*M* change = 54.70%, range = 0–83%) and seven of 10 participants showed this pattern from the dominant to the nondominant language (*M* change = 39.60%, range = 0–83%; see Figure 4). Of the eight participants who were available for the 3- and 6-month follow-up, six and five individuals demonstrated a significant translation effect for cognates in both the dominant and nondominant languages, respectively. At 12 months post-treatment, four of the original seven individuals demonstrated maintenance of a cognate translation effect to the dominant language, with three of the original seven maintaining this pattern of transfer to the nondominant language.

With regard to cross-linguistic translation of noncognates, four of nine individuals showed a significant translation effect from their nondominant to their dominant language (M change = 28.22%, range = 0–83%) and two of 10 showed this pattern from the dominant to the nondominant language (M change = 8.10%, range = −3–47%), following both phases of treatment. A similar pattern of performance was observed at 3 and 6 months post-treatment. At 12 months post-treatment, one of the original four individuals demonstrated maintenance of a noncognate translation effect in the dominant language, with no individuals maintaining this pattern in their nondominant language.

**Figure 5:**
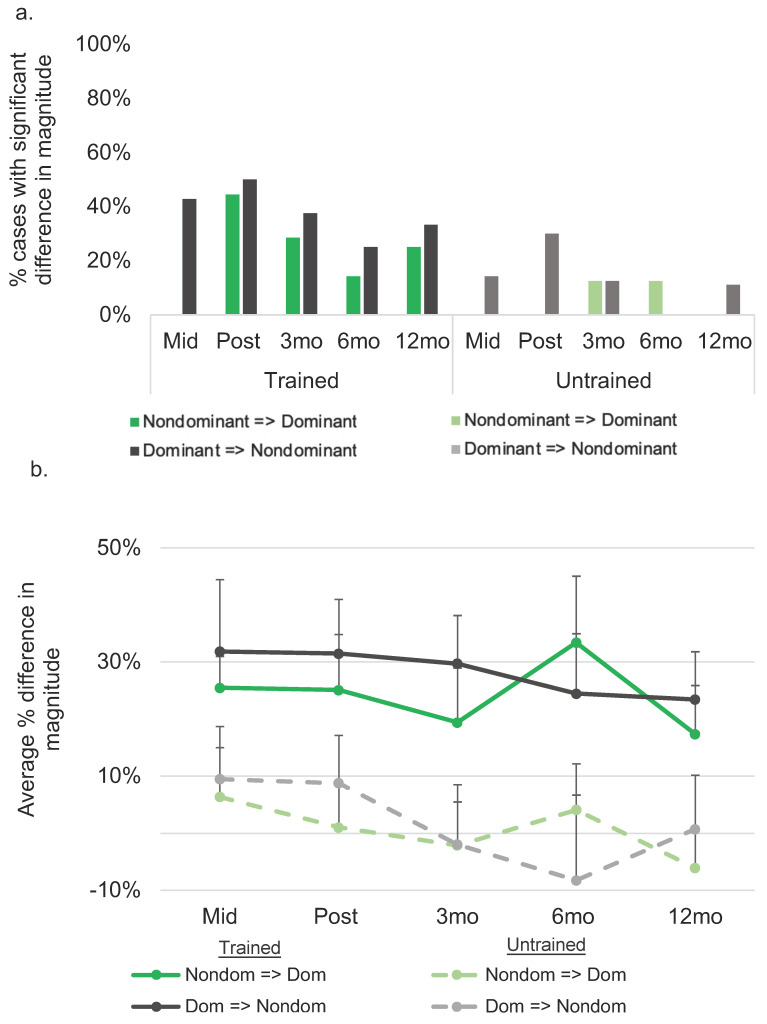
Cross-linguistic transfer for cognates *versus* noncognates at each time point relative to pre-treatment. (**a**). Depicts the percentage of cases demonstrating a significant difference in the magnitude of transfer between cognates and noncognates at each time point relative to pre-treatment. (**b**). Depicts the average difference in the magnitude of change between cognates and noncognates at each time point relative to pre-treatment. Performance on trained items represents translation effects. Performance on untrained items represents generalization effects. At mid-treatment, seven of nine participants had received treatment in their dominant language and two of three who had received treatment in their nondominant language had sufficient data for these contrasts; the figure shows performance for these subsets at mid-tx for trained and untrained items. See Supplemental Materials for data at the single-subject level. Mid = mid-treatment; mo = month.

A direct comparison of the magnitude of translation effects for cognate and noncognate items revealed a significant difference (with better translation of cognates) for four individuals from pre- to post-treatment from the nondominant to the dominant language (M difference = 25.11%, range = −17–83%; see Figure 5), and five participants showing this pattern from their dominant to nondominant language (M difference = 31.50%, range = 0–77%). At subsequent follow-ups, a gradual decline was observed in the number of participants who showed a significant difference in the magnitude of the translation effect observed between cognates and noncognates (see Figure 5).

### 3.4. Performance on Additional Outcome Measures

Paired permutation tests revealed that participants demonstrated significant improvement on the BNT at post-treatment relative to pre-treatment in the nondominant language (*t* = −1.59, *p* = 0.047; see Appendix A). Performance on this measure at other time points was not significantly different from pre-treatment, nor was performance in the dominant language at any time point relative to pre-treatment. Performance on the MMSE showed a relatively steady decline over time, with significant decline emerging at 12 months post-treatment relative to pre-treatment in the dominant language only (*t* = 2.76, *p* = 0.012). Lastly, performance on the WAB-R also showed a gradual decline over time, with significant decline noted at three months post-treatment (*t* = 2.15, *p* = 0.020) and at each subsequent follow-up (6 months post-treatment (*t* = 1.75, *p* = 0.023); one-year post-treatment (*t* = 2.78, *p* = 0.006)) in the dominant language. A similar pattern was observed in the nondominant language, but with significant decline emerging at 6 months post-treatment (*t* = 2.55, *p* = 0.023); one-year post-treatment (t = 2.37; *p* = 0.016).

### 3.5. Self and Communication Partner Assessment of Change

The mean improvement reported by all respondents (caregivers and participants combined) on the post-treatment survey was 1.17 (just above “somewhat better”). The mean rating for participants with lvPPA was 1.68 (between “somewhat better” and better”), and for participants with svPPA, the mean rating was 0.65 (between “unchanged” and “somewhat better”). The average caregiver rating was consistent with the overall mean (1.17). The items and results from the post-treatment survey are reported in Appendix B.

## 4. Discussion

To our knowledge, this is the first study to systematically investigate the utility of speech-language intervention in a group of bilingual speakers with progressive aphasia. Consistent with previous studies examining treatment primarily in monolingual speakers with PPA [14,15], we hypothesized that bilingual speakers would show a robust treatment effect in both of their treated languages, with maintenance at follow-ups. We also hypothesized that generalization to untrained targets would be observed for some participants, due to the strategic nature of the intervention [14,15]. In addition, we sought to investigate whether the inclusion of cross-linguistic cognates would promote accurate translation of treated items.

### 4.1. Within-Language Gains and Generalization Effects

Our results indicate that bilingual speakers with mild-moderate PPA showed a significant and robust treatment effect in both of their treated languages following dual-language naming intervention. With regard to performance on matched, untrained stimuli, a greater number of participants demonstrated generalization in their dominant language at post-treatment; however, generalization was observed in the nondominant language for a smaller subset of participants. This suggests that the strategic nature of the intervention resulted in generalization to untrained items for a subset of participants, with the greatest benefit observed in the dominant language. In sum, our findings constitute further evidence that this treatment approach is beneficial for word retrieval impairments in PPA and FTD. Moreover, our results indicate that this approach is suitable for treating bilingual speakers with progressive anomia, and highlight that significant gains can be observed in both an individual’s dominant and nondominant language.

### 4.2. Maintenance of Treatment Gains

There is pessimism in the clinical and research communities regarding not only the efficacy of treatment in individuals with progressive communication disorders but particularly the potential for maintenance of gains [101]. As such, it is crucial to document not only the immediate benefits of treatment, but also to evaluate stability of treatment effects in the face of disease progression. Many studies that report the effects of intervention in PPA have not explored performance beyond the immediate post-treatment period; however, those that have reported maintenance effects have documented stability in the follow-up period (e.g., [15,38,47,54]). Similarly, stability of treatment effects up to 12 months post-treatment was observed for the majority of our participants. As in Henry et al. (2019), participants were allowed to keep practice materials and encouraged to continue with self- guided practice following the completion of structured intervention with the clinician. In the prior study, post-treatment practice was monitored via self-report for a subset of participants and, surprisingly, a relation was not observed between amount of ongoing practice and maintenance of treatment gains. Future studies should employ methods for systematic and objective tracking of individual practice to better understand maintenance effects and the role of continued practice for individuals with PPA [15].

The maintenance effects in this study can be interpreted within the broader context of cognitive-linguistic decline observed in this cohort of bilingual speakers. Specifically, participants demonstrated gradual decline on general measures of linguistic and cognitive functioning (see Appendix A). In the context of this general progression, our findings confirm that a tailored approach to bilingual intervention results in significant improvement for trained items as well as improvement or stability in confrontation naming more broadly (as noted on the BNT, see Supplementary Materials). These findings indicate a possible protective benefit for the targeted behavior following treatment.

### 4.3. Cross-Linguistic Translation Effects

We observed that the majority of individuals showed a significant cognate translation effect (i.e., ability to name cognate items in the untrained language) following both phases of treatment, with fewer individuals showing an effect following the initial phase of treatment (see the Supplementary Materials for results following the initial treatment phase). For approximately half of participants, the translation effect at post-treatment was significantly greater in magnitude for cognates relative to noncognates. Cognate translation effects were generally maintained up to six months post-treatment (consistent with within-language generalization observed in our prior study [15]), with fewer individuals demonstrating a sustained benefit one year post-treatment. We note a couple of interesting patterns that emerged from our data. First, the two individuals who did not show a cognate translation effect (in at least one linguistic direction) obtained the lowest cognitive screening score (MMSE; lv3) or naming score (BNT; sv3) at pre-treatment. This observation suggests that an individual’s potential to benefit from inclusion of cognates may be mediated by severity of cognitive and/or language deficits. This is also consistent with the finding that the most notable decrease in cognate translation ability (for individuals who originally showed a cognate translation effect) occurred between the 6 and 12-month follow-up visits (i.e., with increasing severity of cognitive-linguistic deficits). 

Pre- and postmorbid language history variables, such as order of acquisition and frequency of use, may also influence translation effects (e.g., [3,4,5,68]). In PPA and other neurodegenerative disorders, nonparallel patterns of language decline [6,99,100,101] have been reported, which may influence frequency of language use and moderate treatment outcomes across languages. In the future, larger samples will allow us to better understand the relation between overall severity and translation effects, and to explore the possible interaction of severity indices and language history variables.

Given that the distribution of participants who received treatment in the dominant vs. nondominant language during the initial phase is unbalanced in this study (*n* = 3 received treatment in nondominant language in the initial phase), the following preliminary observations should be interpreted with caution and require replication in a larger sample utilizing a balanced design. Following the first treatment phase, a greater proportion of participants showed a cognate translation effect from the nondominant to dominant language (i.e., three of three participants who were treated in the nondominant language in the initial phase and three of seven who were treated in the dominant language in the initial phase). Findings following the initial phase of treatment are consistent with (1) patterns observed in healthy bilingual speakers (e.g., [62,66]) and with (2) transfer and translation patterns observed in stroke-induced aphasia (e.g., [65,82]), wherein ease of translation may be facilitated from the weaker to stronger language. Following both phases of treatment, the cross-linguistic translation effects for cognate items were bidirectional in our cohort, (i.e., eight of 10 from the nondominant to the dominant language and seven of 10 participants from the dominant to the nondominant language). This may indicate that the treatment approach, which targets both semantic and phonological bases of word retrieval, strengthened cross-linguistic activation between translation equivalents with phonological similarities. Together, results indicate that treatment in the nondominant language (or treatment in the dominant language followed by treatment in the nondominant language) resulted in robust translation effects for cognate items. We reiterate that this observation should be interpreted with caution due to the small number of participants, heterogeneity in clinical profile, and crucially, the fact that fewer participants received treatment in their nondominant language in the initial phase of this study (*n* = 3).

It is important to note that noncognates also have the potential to benefit cross-linguistically from this intervention, due to the targeted analysis of semantic features, which are shared across languages [58,102,103]. Nonetheless, far fewer individuals showed a significant noncognate translation effect after the initial phase of treatment (i.e., three of three from the nondominant to the dominant language and zero of seven from the dominant to the nondominant language) or following both phases of treatment (i.e., four of nine from their nondominant to their dominant language and two of 10 from their dominant to their nondominant language). The diminished translation effects for noncognates relative to cognates may be driven by a lack of phonological similarity. This is corroborated by findings from healthy bilingual speakers, which suggest that the combination of shared conceptual representations and phonology leads to the well-documented cognate facilitation effect (e.g., [56,57,60,61,62,63,64,65,66,67,104]). 

### 4.4. Treatment and Translation Effects by PPA Variant

Following both phases of treatment, we observed that individuals with either sv or lvPPA showed a significant treatment effect irrespective of language dominance. With regard to generalization to untrained items, a slightly greater number of individuals within each variant showed generalization in the dominant language (sv = 3 vs. 1; lv = 4 vs. 3).

Although we did not have specific hypotheses regarding treatment and translation effects on the basis of the PPA variant, in this section, we note patterns that emerged in this study. With respect to cross-linguistic translation effects, individuals with svPPA and those with lvPPA demonstrated evidence of cognate translation effects. By contrast, a subset (four of nine from their nondominant to their dominant language and two of 10 from their dominant to their nondominant language) of individuals with lvPPA and no individuals with svPPA demonstrated significant translation effects for noncognates. This pattern may be explained by the different underlying deficits contributing to naming impairment in each variant. In lvPPA, semantic processing is relatively spared, and cognate facilitation is likely a result of improved access to or assembly of phonology resulting from repeated practice of target items. In the case of noncognates, the translation effects in some lvPPA cases may be attributed to the strategic nature of the intervention, which requires individuals to use residual semantic and word form knowledge in attempts to self-cue. In lvPPA, translation of noncognates was greatest from the nondominant to the dominant language. As has been reported in bilingual AD (e.g., [105,106]), it may be the case that the dominant language is more resistant to decline in bilingual speakers with lvPPA [107], and perhaps more likely to benefit from translation effects. This pattern might also reflect reliance upon the dominant language to access semantic knowledge [58].

In svPPA, learning has been characterized as rigid, with generalization reported less frequently (e.g., [37,38,39,43,108,109]). In addition, phonological processing is relatively spared [107] and cognate translation effects may be facilitated by strengthening of semantic representations for trained items, with similarities in phonology boosting activation for these word forms across languages. Given that learning tends to be more rigid in svPPA, it is not surprising that significant translation effects for noncognates (where spared phonological processing would not confer the same benefit) were not observed.

### 4.5. Additional Considerations

This study provides evidence that lexical retrieval treatment is an efficacious intervention for bilingual speakers with PPA in the mild-to-moderate range of severity. All individuals in this study were seen via a telehealth platform, which allowed for the inclusion of individuals living throughout the United States, as well as internationally. Telehealth holds promise as an assessment and treatment modality, enabling clinicians to reach individuals who may face barriers to accessing treatment, including ethnically and racially diverse groups who experience barriers to service provision more generally (e.g., [110,111,112]). In the future, advocacy for broad reimbursement of these services will be crucial to exploiting this treatment modality. In addition, future research should continue to broaden the evidence base for telehealth interventions intended for individuals with PPA beyond the mild-to-moderate range in order to maximize communication across the continuum of disease severity.

This study had several limitations. First, although this is, to our knowledge, the largest intervention study of bilingual speakers with PPA and FTD to date, the sample size is a limiting factor. Future studies will benefit from larger samples in order to investigate patterns of response to treatment, including cross-linguistic effects. This will also allow for the investigation of different patterns on the basis of language distance (i.e., how similar language pairs are to one another), as well as the consideration of language history variables (e.g., age of acquisition, frequency of use). It is also important to note that our results represent findings from language pairs that share cross-linguistic cognates. For individuals who speak language pairs that do not share cognates, our results suggest that the strategic component of this intervention may encourage generalization to untrained items and that cross-linguistic transfer is possible for noncognate items (particularly for individuals with lvPPA).

The treatment approach used in this study was selected due to its established benefit in monolingual speakers with PPA and due to its emphasis on training procedures that draw upon both semantic and phonological mechanisms supporting naming. From this study, it is not possible to discern whether semantic versus phonological stimulation is more crucial for within-language outcomes and transfer effects in this population. Future research may employ facilitation studies to investigate whether particular components of intervention are especially supportive of translation and generalization effects in bilingual speakers with progressive anomia. In addition, there is a need to investigate the potential for generalized improvement from naming intervention to connected speech, as such effects would further characterize the ecological validity of naming interventions administered to individuals with PPA.

## 5. Conclusions

There is a growing literature base addressing the treatment of progressive disorders of language. This work is crucial, as the global community anticipates a rapidly growing aging population, and consequently, an increase in the number of individuals presenting with neurodegenerative disorders (e.g., [113,114]). Simultaneously, we anticipate a growing bilingual population [115,116]. Although previous work has established a strong foundation for speech-language treatment research in monolingual speakers with neurodegenerative disorders, much work is needed to address the optimization of these approaches for bilingual speakers. At the same time, careful consideration of assessment and treatment methods is needed to ensure the use of culturally tailored approaches, as bilingual speakers often comprise culturally and ethnically diverse groups [12,117].

Our results indicate that bilingual speakers with PPA and FTD significantly improved their word retrieval for trained items assigned to each of their languages, with maintenance observed up to 6 or 12 months post-treatment. In addition, our findings indicate that monolingual clinicians may be able to select cross-linguistic cognates as a means to support gains across languages for words trained in a single language (i.e., “two for the price of one”). This has ramifications for service delivery in the U.S., where a majority of clinicians are monolingual English speakers. In the context of results from previous studies investigating treatment outcomes for PPA, our results offer complementary support and confirm that tailored behavioral intervention should be the standard of clinical care for linguistically diverse individuals with progressive aphasia.

## Figures and Tables

**Table 1:** Individual Demographic and Language History Profiles.

**Participant**	rtFTD1		SV1		SV2		SV3		SV4		LV1		LV2		LV3		LV4		LV5	
**Demographics**																				
Sex	M		M		M		F		F		F		M		F		M		M	
Age (years)	67		72		64		60		63		78		80		59		64		62	
Education (years)	16		12		18		20		20		16		20		18		18		13	
Years Post Onset	3		5		3		3		9		2		4		2		2.5		1.5	
Handedness	Right		Right		Right		Ambidextrous		Right		Right		Right		Right		Right		Right	
**Language History Variables**																				
**Language**	Span	Eng	Span	Eng	Farsi	Eng	Span	Eng	Span	Eng	French	Eng	Port	Eng	Span	Eng	Farsi	Eng	Span	Eng
Age of acquisition (years)	Birth	6	Birth	5	Birth	18	11	Birth	9	Birth	17	Birth	Birth	Birth	Birth	16	Birth	14	Birth	17
Premorbid proficiency (5-point scale; with 5 indicating native-like proficiency)	3	5	4	5	4	5	3	5	5	5	5	5	4	5	5	5	5	4	5	5
Premorbid daily usage (out of 100%)	7%	93%	16%	85%	60%	40%	8%	93%	12%	88%	13%	88%	10%	90%	20%	80%	37%	63%	48%	52%
Weekday	13%	87%	18%	82%	38%	62%	15%	85%	12%	88%	13%	88%	13%	87%	20%	80%	53%	47%	46%	54%
Weekend	1%	99%	13%	87%	82%	18%	0%	100%	12%	88%	13%	88%	6%	94%	20%	80%	21%	79%	50%	50%
Postmorbid proficiency (5-point scale; with 5 indicating native-like proficiency)	3	5	3	5	4	5	2	4	2	3	5	5	2	4	2	2	5	4	3	4
Postmorbid daily usage (out of 100%)	5%	94%	7%	93%	60%	40%	8%	93%	97%	3%	13%	88%	6%	94%	10%	90%	80%	20%	85%	15%
Weekday	6%	91%	7%	93%	38%	62%	15%	85%	100%	0%	13%	88%	6%	94%	10%	90%	80%	20%	87%	13%
Weekend	3%	97%	7%	93%	82%	18%	0%	100%	94%	6%	13%	88%	6%	94%	10%	90%	80%	20%	83%	17%
Self-reported dominance	English		English		Farsi		English		English		English		English		English		Farsi		Spanish	
Dominance index (lower BNT score/ higher BNT score)	0.39		0.15		0.50		0.33		0.82		0.50		0.14		0.22		0.47		0.97	

Note: rtFTD1 = participant with right temporal variant frontotemporal dementia; sv = semantic variant PPA; lv = logopenic variant PPA. See Gollan et al., 2010; 2012 for details regarding dominance index. Span = Spanish, Eng = English, Fre = French, Port = Portuguese.

**Table 2:** Pre-Treatment Assessment Battery.

Participant ID	rtFTD1		SV1		SV2		SV3		SV4		LV1		LV2		LV3		LV4		LV5	
Language	Span	Eng	Span	Eng	Farsi	Eng	Span	Eng	Span	Eng	Fre	Eng	Port	Eng	Spa	Eng	Farsi	Eng	Span	Eng
Mini-Mental State Examination ^1^ (30)	23	22	15	25	30	27	17	23	14	17	23	26	6	29	9	14	29	27	26	27
CVLT Total (36) ^2^	15	16	13	18	-	15	0	13	-	13	-	19	-	17	-	11	-	24	9	11
CVLT 10-min Recall ^2^	1	3	0	0	-	0	0	0	-	1	-	5	-	2	-	3	-	3	0	0
Stroop Color naming ^2^	26	38	12	45	-	38	-	35	11	48	-	42	-	52	-	7	-	69	38	38
Stroop interference ^2^	14	24	7	30	-	12	-	21	9	31	-	31	-	20	-	4	-	49	23	22
Complex Figure Copy (17) ^2^	-	14	-	14	-	15	-	17	-	17	-	13	-	15	-	7	-	16	16	-
Complex Figure Recall (17) ^2^	-	6	-	3	-	13	-	15	-	11	-	10	-	6	-	4	-	17	5	-
Calculations (5) ^2^	-	5	-	4	-	5	-	-	-	-	-	3	-	-	-	0	-	5	-	-
Digit Span Forward ^2^	4	5	5	6	-	6	5	7	4	6	-	6	-	5	-	3	-	6	3	3
Digit Span Backward ^2^	3	4	3	5	-	5	4	5	5	5	-	4	-	4	-	2	-	4	4	3
PPVT Short (16) ^2^	-	14	-	10	-	8	-	1	-	4	-	-	-	12	-	9	-	8	-	13
Western Aphasia Battery (AQ; 100) ^3^	78.2	92.6	69.2	87.5	90.2	81.3	42.9	75.9	51	74.4	77.3	88.7	38.4	86.8	39.3	61.3	92	82.1	84.4	82.8
Motor Speech Eval: AOS (0–7) ^4^	0	0	0	0	-	0	N/A	N/A	-	0	-	0	-	0	-	0	-	0	0	0
Motor Speech Eval: Dysarthria (0–7) ^4^	0	0	0	0	-	0	N/A	N/A	-	0	-	0	-	0	-	0	-	0	0	0
Pyramids and Palm Trees Test ^5^ (short; 14 ^6^; * = /25, ^ = /20 ^7^)	-	14	-	14	12	14	-	7	14 ^	14 *	-	13	-	14	-	13	-	14	-	13
Boston Naming Test (60; * = /18) ^8^	11	28	4	27	8	4	1	3	2 *	2	17	34	4	29	2	9	43	20	33	34
UCSF Syntax Comprehension Test (%) ^9^	-	97	-	100	-	97	-	-	-	-	-	97	-	100	-	75	-	92	-	-
BAT Syntax Comprehension Subtest (%) ^10^	92	100	79	98	93	92	69	95	84	84	74	91	76	91	51	53	94	92	8	85
Arizona Phonological Battery (%) ^11^	-	50	-	80	-	53	-	97	-	94	-	58	-	56	-	8	-	69	-	50

Note: rtFTD1 = participant with right temporal variant frontotemporal dementia; sv = semantic variant PPA; lv = logopenic variant PPA, BAT = Bilingual Aphasia Test, Span = Spanish, Eng = English, Fre = French, Port = Portuguese. ^1^ Folstein, Folstein & McHugh, 1975; ^2^ Kramer et al., 2003; ^3^ Kertesz, 1982; ^4^ Wertz, LaPointe & Rosenbek, 1984; ^5^ Howard & Patterson, 1982; ^6^ Breining et al., 2015; ^7^ Martínez-Cuitiño & Barreyro, 2010; ^8^ Kaplan, Goodglass & Weintraub, 2001; ^9^ Wilson, Dronkers, et al., 2010; ^10^ Paradis & Libben, 1987; ^11^ Beeson et al., 2010.

**Table 3:** Lexical Retrieval Cascade Used During Treatment Sessions (Henry et al., 2013; 2019).

1. (Picture is presented) Semantic self-cue	Clinician prompts semantic description with, “Tell me about it.” Additional prompting follows, as needed: “Where would you find this? What is it used for? Do you have any memories about this?” (If the item is named in this step, the clinician proceeds to step 5.)
2. Orthographic self-cue	Clinician requests written form of the word: “Can you write the word?” If unable to, the participant is encouraged to think of the first letter and/or sound of the word and any other characteristics about the word (i.e., “Is it a long or a short word?”). If the participant cannot come up with the first letter, the clinician writes the first grapheme.
3. Phonemic self-cue	Clinician asks the participant to make the sound associated with the letter. (If the item is named in this step, the clinician proceeds to step 5.)
4. Oral reading	If the item is not yet named, the clinician writes out the remainder of the word and the participant reads it aloud.
5. Written and Spoken Repetition	The participant writes and says the word three times.
6. Semantic Plausibility Judgments	Clinician asks three yes/no questions regarding semantic features of the item (e.g., “would you find this in a toolbox?”)
7. Recall	Clinician asks the participant to provide the most salient semantic features and write and say the word one time.

**Figure A1:**
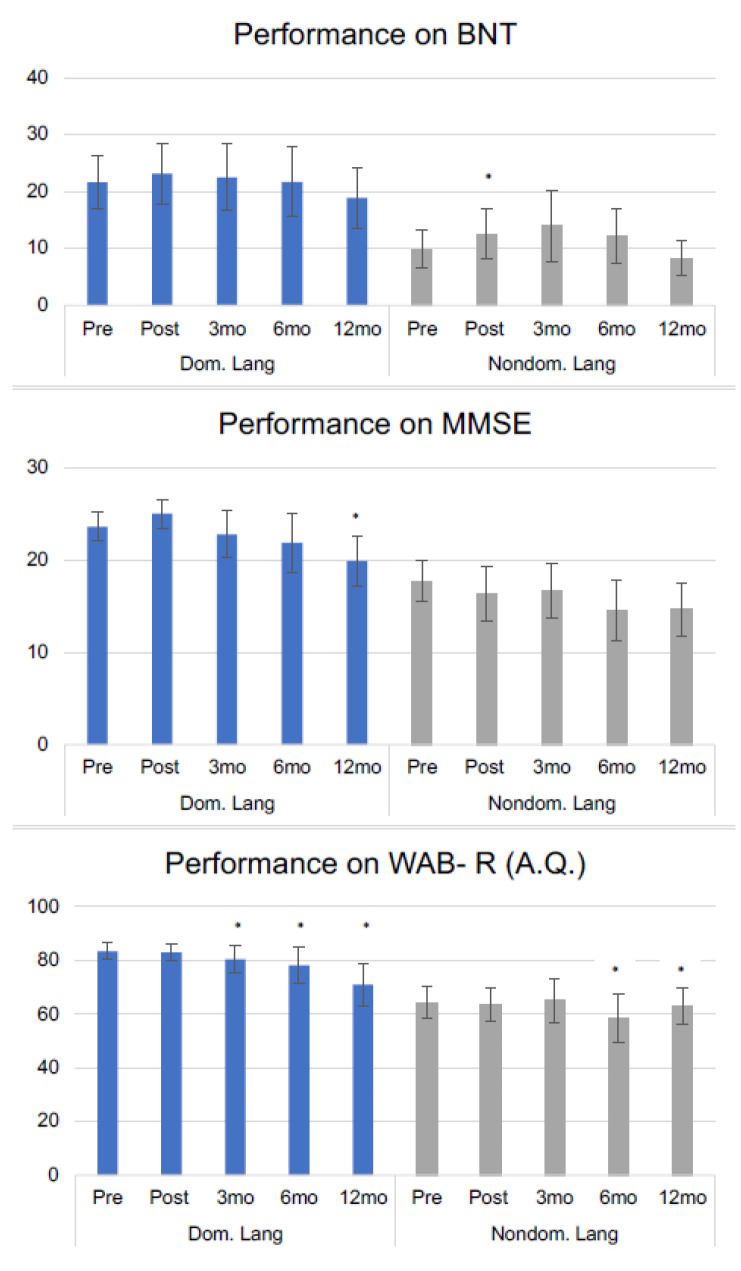
Performance from pre-treatment to each subsequent timepoint on a subset of cognitive and linguistic measures by language dominance. * Note. Standard error bars included for descriptive purposes. Significance determined via paired permutation tests. One-tailed tests used for BNT from pre to post-treatment; two-tailed tests used for all other timepoints/measures (*p* < 0.05). Dom. = dominant language, Nondom. = nondominant language, BNT = Boston Naming Test; MMSE = Mini-Mental State Exam, WAB-R = Western Aphasia Battery- Revised.

**Figure A2:**
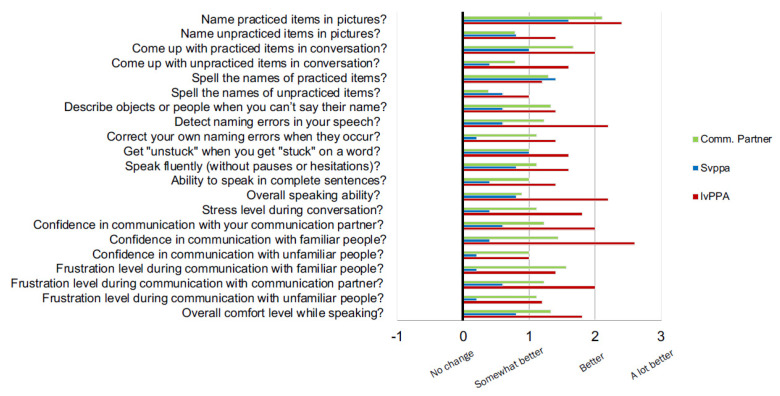
Survey Responses: “Compared to pre-treatment how would you rate your ability to…”.

## Data Availability

The data presented in this study are available in the supplemental materials.

## Supplementary Materials

The following are available online at https://www.mdpi.com/article/10.3390/brainsci11111371/s1, Table S1: Results of Statistical Analyses at the Single-Subject Level for all Participants, Text S1: Results Following the Initial Phase of Treatment and Cross-Linguistic Generalization Effects to Untrained Items.

## References

[1] Grosjean F. (2010). Bilingual: Life and Reality.

[2] Marian V., Shook A. (2012). The Cognitive Benefits of Being Bilingual. Cerebrum Dana Forum Brain Sci..

[3] Faroqi-Shah Y., Frymark T., Mullen R., Wang B. (2010). Effect of treatment for bilingual individuals with aphasia: A systematic review of the evidence. J. Neurolinguist..

[4] Kohnert K. (2009). Cross-Language Generalization following Treatment in Bilingual Speakers with Aphasia: A Review. Semin. Speech Lang..

[5] Ansaldo A.I., Saidi L.G. (2014). Aphasia Therapy in the Age of Globalization: Cross-Linguistic Therapy Effects in Bilingual Aphasia. Behav. Neurol..

[6] Sandberg C.W., Zacharewicz M., Gray T. (2021). Bilingual Abstract Semantic Associative Network Training (BAbSANT): A Polish-English case study. J. Commun. Disord..

[7] Peñaloza C., Scimeca M., Gaona A., Carpenter E., Mukadam N., Gray T., Shamapant S., Kiran S. (2021). Telerehabilitation for Word Retrieval Deficits in Bilinguals With Aphasia: Effectiveness and Reliability as Compared to In-person Language Therapy. Front. Neurol..

[8] Aziz M.A.A., Razak R.A., Garraffa M. (2020). Targeting Complex Orthography in the Treatment of a Bilingual Aphasia with Acquired Dysgraphia: The Case of a Malay/English Speaker with Conduction Aphasia. Behav. Sci..

[9] Costa A.S., Jokel R., Villarejo A., Llamas-Velasco S., Domoto-Reilley K., Wojtala J., Reetz K., Machado Á. (2019). Bilingualism in Primary Progressive Aphasia. Alzheimer Dis. Assoc. Disord..

[10] Meyer A.M., Snider S.F., Eckmann C.B., Friedman R.B. (2015). Prophylactic treatments for anomia in the logopenic variant of primary progressive aphasia: Cross-language transfer. Aphasiology.

[11] Rumbaut R.G., Massey D.S. (2013). Immigration & Language Diversity in the United States. Daedalus.

[12] Santhanam S.P., Parveen S. (2018). Serving Culturally and Linguistically Diverse Clients: A Review of Changing Trends in Speech-Language Pathologists’ Self-efficacy and Implications for Stakeholders. Clin. Arch. Commun. Disord..

[13] ASHA (2020). Demographic Profile of ASHA Members Providing Bilingual Services.

[14] Henry M., Rising K., DeMarco A., Miller B., Gorno-Tempini M., Beeson P. (2013). Examining the value of lexical retrieval treatment in primary progressive aphasia: Two positive cases. Brain Lang..

[15] Henry M.L., Hubbard H.I., Grasso S.M., Dial H.R., Beeson P.M., Miller B.L., Gorno-Tempini M.L. (2019). Treatment for Word Retrieval in Semantic and Logopenic Variants of Primary Progressive Aphasia: Immediate and Long-Term Outcomes. J. Speech Lang. Hear. Res..

[16] Beeson P.M., King R.M., Bonakdarpour B., Henry M.L., Cho H., Rapcsak S.Z. (2011). Positive Effects of Language Treatment for the Logopenic Variant of Primary Progressive Aphasia. J. Mol. Neurosci..

[17] Mesulam M.-M. (1982). Slowly progressive aphasia without generalized dementia. Ann. Neurol..

[18] Gorno-Tempini M.L., Hillis A.E., Weintraub S., Kertesz A., Mendez M., Cappa S.F., Ogar J.M., Rohrer J.D., Black S., Boeve B.F. (2011). Classification of primary progressive aphasia and its variants. Neurology.

[19] Montembeault M., Brambati S.M., Gorno-Tempini M.L., Migliaccio R. (2018). Clinical, Anatomical, and Pathological Features in the Three Variants of Primary Progressive Aphasia: A Review. Front. Neurol..

[20] Henry M.L., Gorno-Tempini M.L. (2010). The logopenic variant of primary progressive aphasia. Curr. Opin. Neurol..

[21] Rohrer J., Rossor M., Warren J.D. (2012). Alzheimer’s pathology in primary progressive aphasia. Neurobiol. Aging.

[22] Spinelli E.G., Mandelli M.L., Miller Z.A., Santos-Santos M.A., Wilson S.M., Agosta F., Grinberg L.T., Huang E.J., Trojanowski J.Q., Meyer M. (2017). Typical and atypical pathology in primary progressive aphasia variants. Ann. Neurol..

[23] Hodges J.R., Patterson K. (2007). Semantic dementia: A unique clinicopathological syndrome. Lancet Neurol..

[24] Iaccarino L., Crespi C., Della Rosa P.A., Catricalà E., Guidi L., Marcone A., Tagliavini F., Magnani G., Cappa S., Perani D. (2015). The Semantic Variant of Primary Progressive Aphasia: Clinical and Neuroimaging Evidence in Single Subjects. PLoS ONE.

[25] Binney R.J., Henry M., Babiak M., Pressman P.S., Santos-Santos M.A., Narvid J., Mandelli M.L., Strain P.J., Miller B.L., Rankin K.P. (2016). Reading words and other people: A comparison of exception word, familiar face and affect processing in the left and right temporal variants of primary progressive aphasia. Cortex.

[26] Josephs K.A., Whitwell J.L., Knopman D.S., Boeve B.F., Vemuri P., Senjem M.L., Parisi J.E., Ivnik R.J., Dickson D.W., Petersen R.C. (2009). Two distinct subtypes of right temporal variant frontotemporal dementia. Neurology.

[27] Chan D., Anderson V., Pijnenburg Y., Whitwell J., Barnes J., Scahill R., Stevens J.M., Barkhof F., Scheltens P., Rossor M. (2009). The clinical profile of right temporal lobe atrophy. Brain.

[28] Henry M.L., Wilson S.M., Ogar J.M., Sidhu M.S., Rankin K.P., Cattaruzza T., Miller B.L., Gorno-Tempini M.L., Seeley W.W. (2014). Neuropsychological, behavioral, and anatomical evolution in right temporal variant frontotemporal dementia: A longitudinal and post-mortem single case analysis. Neurocase.

[29] Carthery-Goulart M.T., Silveira A.D.C.D., Machado T.H., Mansur L.L., Parente M.A.D.M.P., Senaha M.L.H., Brucki S., Nitrini R. (2013). Nonpharmacological interventions for cognitive impairments following primary progressive aphasia: A systematic review of the literature. Dement. Neuropsychol..

[30] Croot K., Nickels L., Laurence F., Manning M. (2009). Impairment- and activity/participation-directed interventions in progressive language impairment: Clinical and theoretical issues. Aphasiology.

[31] Jokel R., Graham N.L., Rochon E., Leonard C. (2014). Word retrieval therapies in primary progressive aphasia. Aphasiology.

[32] Rising K. (2016). Treatment for Lexical Retrieval in Primary Progressive Aphasia Lexical Retrieval Treatments in PPA. Perspect. Neurophysiol. Neurogenic Speech Lang. Disord..

[33] Tippett D.C., Hillis A.E., Tsapkini K. (2015). Treatment of Primary Progressive Aphasia. Curr. Treat. Options Neurol..

[34] Croot K. (2018). Treatment for Lexical Retrieval Impairments in Primary Progressive Aphasia: A Research Update with Implications for Clinical Practice. Semin. Speech Lang..

[35] Cadório I., Lousada M., Martins P., Figueiredo D. (2017). Generalization and maintenance of treatment gains in primary progressive aphasia (PPA): A systematic review. Int. J. Lang. Commun. Disord..

[36] Cotelli M., Manenti R., Ferrari C., Gobbi E., Macis A., Cappa S.F. (2020). Effectiveness of language training and non-invasive brain stimulation on oral and written naming performance in Primary Progressive Aphasia: A meta-analysis and systematic review. Neurosci. Biobehav. Rev..

[37] Graham K.S., Patterson K., Pratt K.H., Hodges J.R. (1999). Relearning and Subsequent Forgetting of Semantic Category Exemplars in a Case of Semantic Dementia. Neuropsychology.

[38] Heredia C.G., Sage K., Ralph M.A.L., Berthier M.L. (2009). Relearning and retention of verbal labels in a case of semantic dementia. Aphasiology.

[39] Mayberry E., Sage K., Ehsan S., Ralph M.A.L. (2011). Relearning in semantic dementia reflects contributions from both medial temporal lobe episodic and degraded neocortical semantic systems: Evidence in support of the complementary learning systems theory. Neuropsychologia.

[40] Jokel R., Rochon E., Anderson N. (2010). Errorless learning of computer-generated words in a patient with semantic dementia. Neuropsychol. Rehabil..

[41] Savage S., Ballard K., Piguet O., Hodges J.R. (2013). Bringing words back to mind—Improving word production in semantic dementia. Cortex.

[42] Savage S.A., Piguet O., Hodges J.R. (2015). Cognitive Intervention in Semantic Dementia Maintaining Words over Time. Alzheimer Dis. Assoc. Disord..

[43] Snowden J.S., Neary D. (2002). Relearning of verbal labels in semantic dementia. Neuropsychologia.

[44] Suarez-Gonzalez A., Heredia C.G., Savage S.A., Gil-Néciga E., García-Casares N., Franco-Macías E., Berthier M.L., Caine D. (2014). Restoration of conceptual knowledge in a case of semantic dementia. Neurocase.

[45] Suárez-González A., A Savage S., Caine D. (2016). Successful short-term re-learning and generalisation of concepts in semantic dementia. Neuropsychol. Rehabil..

[46] Meyer A.M., Tippett D.C., Friedman R.B. (2016). Prophylaxis and remediation of anomia in the semantic and logopenic variants of primary progressive aphasia. Neuropsychol. Rehabil..

[47] Croot K., Raiser T., Taylor-Rubin C., Ruggero L., Ackl N., Wlasich E., Danek A., Scharfenberg A., Foxe D., Hodges J.R. (2019). Lexical retrieval treatment in primary progressive aphasia: An investigation of treatment duration in a heterogeneous case series. Cortex.

[48] Lavoie M., Bier N., Laforce R., Macoir J. (2020). Improvement in functional vocabulary and generalization to conversation following a self-administered treatment using a smart tablet in primary progressive aphasia. Neuropsychol. Rehabil..

[49] Krajenbrink T., Croot K., Taylor-Rubin C., Nickels L. (2018). Treatment for spoken and written word retrieval in the semantic variant of primary progressive aphasia. Neuropsychol. Rehabil..

[50] Dressel K., Huber W., Frings L., Kümmerer D., Saur D., Mader I., Hüll M., Weiller C., Abel S. (2010). Model-oriented naming therapy in semantic dementia: A single-case fMRI study. Aphasiology.

[51] Jokel R., Anderson N. (2012). Quest for the best: Effects of errorless and active encoding on word re-learning in semantic dementia. Neuropsychol. Rehabil..

[52] Newhart M., Davis C., Kannan V., Heidler-Gary J., Cloutman L., Hillis A.E. (2009). Therapy for naming deficits in two variants of primary progressive aphasia. Aphasiology.

[53] Grasso S.M., Shuster K.M., Henry M.L. (2017). Comparing the effects of clinician and caregiver-administered lexical retrieval training for progressive anomia. Neuropsychol. Rehabil..

[54] Meyer A.M., Tippett D.C., Turner R.S., Friedman R.B. (2019). Long-Term maintenance of anomia treatment effects in primary progressive aphasia. Neuropsychol. Rehabil..

[55] Beales A., Cartwright J., Whitworth A., Panegyres P.K. (2016). Exploring generalisation processes following lexical retrieval intervention in primary progressive aphasia. Int. J. Speech-Lang. Pathol..

[56] Kroll J., Stewart E. (1994). Category Interference in Translation and Picture Naming: Evidence for Asymmetric Connections between Bilingual Memory Representations. J. Mem. Lang..

[57] Kroll J.F., Michael E., Tokowicz N., Dufour R. (2002). The development of lexical fluency in a second language. Second. Lang. Res..

[58] Kroll J.F., Van Hell J.G., Tokowicz N., Green D.W. (2010). The Revised Hierarchical Model: A critical review and assessment. Biling. Lang. Cogn..

[59] van Hell J.G., de Groot A.M. (2008). Sentence context modulates visual word recognition and translation in bilinguals. Acta Psychol..

[60] Christoffels I., De Groot A., Kroll J. (2006). Memory and language skills in simultaneous interpreters: The role of expertise and language proficiency. J. Mem. Lang..

[61] De Groot A.M.B. (1992). Determinants of word translation. J. Exp. Psychol. Learn. Mem. Cogn..

[62] DeGroot A., Dannenburg L., Vanhell J. (1994). Forward and Backward Word Translation by Bilinguals. J. Mem. Lang..

[63] de Groot A.M.B., Nas G.L.J. (1991). Lexical Representation of Cognates and Noncognates Compound Bilinguals. J. Mem. Lang..

[64] Costa A., Caramazza A., Sebastian-Galles N. (2000). The Cognate Facilitation Effect: Implications for Models of Lexical Access. J. Exp. Psychology Learn. Mem. Cogn..

[65] Costa A., Santesteban M., Caño A. (2005). On the facilitatory effects of cognate words in bilingual speech production. Brain Lang..

[66] Sáchez-Casas R.M., García-Albea J.E., Davis C.W. (1992). Bilingual lexical processing: Exploring the cognate/non-cognate distinction. Eur. J. Cogn. Psychol..

[67] Rosselli M., Ardila A., Jurado M.B., Salvatierra J.L. (2014). Cognate facilitation effect in balanced and non-balanced Spanish–English bilinguals using the Boston Naming Test. Int. J. Biling..

[68] Murray L.L. (2014). Bilingual aphasia treatment: Clinical recommendations regarding secondary language treatment, cross-language transfer, and the use of language brokers await additional research. Evid.-Based Commun. Assess. Interv..

[69] Hameau S., Köpke B. (2015). Cross-Language Transfer for Cognates in Aphasia Therapy with Multilingual Patients: A Case Study. Aphasie Verwandte Geb..

[70] Marangolo P., Rizzi C., Peran P., Piras F., Sabatini U. (2009). Parallel recovery in a bilingual aphasic: A neurolinguistic and fMRI study. Neuropsychology.

[71] Miertsch B., Meisel J.M., Isel F. (2009). Non-treated languages in aphasia therapy of polyglots benefit from improvement in the treated language. J. Neurolinguist..

[72] Kiran S., Sandberg C., Gray T., Ascenso E., Kester E. (2013). Rehabilitation in Bilingual Aphasia: Evidence for Within- and Between-Language Generalization. Am. J. Speech-Lang. Pathol..

[73] Kiran S., Iakupova R. (2011). Understanding the relationship between language proficiency, language impairment and rehabilitation: Evidence from a case study. Clin. Linguist. Phon..

[74] Ansaldo A.I., Saidi L.G., Ruiz A. (2009). Model-driven intervention in bilingual aphasia: Evidence from a case of pathological language mixing. Aphasiology.

[75] Edmonds L.A., Kiran S. (2006). Effect of Semantic Naming Treatment on Crosslinguistic Generalization in Bilingual Aphasia. J. Speech Lang. Hear. Res..

[76] Junqué C., Vendrell P., Vendrell-Brucet J.M., Tobena A. (1989). Differential recovery in naming in bilingual aphasics. Brain Lang..

[77] Kiran S., Roberts P.M. (2009). Semantic feature analysis treatment in Spanish–English and French–English bilingual aphasia. Aphasiology.

[78] Kohnert K. (2004). Cognitive and cognate-based treatments for bilingual aphasia: A case study. Brain Lang..

[79] Goral M., Rosas J., Conner P.S., Maul K.K., Obler L.K. (2012). Effects of language proficiency and language of the environment on aphasia therapy in a multilingual. J. Neurolinguist..

[80] Meinzer M., Obleser J., Flaisch T., Eulitz C., Rockstroh B. (2007). Recovery from aphasia as a function of language therapy in an early bilingual patient demonstrated by fMRI. Neuropsychologia.

[81] Kurland J., Falcon M. (2011). Effects of cognate status and language of therapy during intensive semantic naming treatment in a case of severe nonfluent bilingual aphasia. Clin. Linguist. Phon..

[82] Bedore L.M., Peña E.D., García M., Cortez C. (2005). Scoring: When Does It Make a Difference?. Lang. Speech Hear. Serv. Sch..

[83] Kohnert K.J., Hernandez A.E., Bates E. (1998). Bilingual Performance on the Boston Naming Test: Preliminary Norms in Spanish and English. Brain Lang..

[84] Umbel V.M., Pearson B.Z., Fernandez M.C., Oller D.K. (1992). Measuring Bilingual Children’s Receptive Vocabularies. Child Dev..

[85] Folstein M.F., Folstein S.E., McHugh P.R. (1975). “Mini-mental state”: A practical method for grading the cognitive state of patients for the clinician. J. Psychiatr. Res..

[86] Dial H.R., A Hinshelwood H., Grasso S.M., Hubbard H.I., Gorno-Tempini M.-L., Henry M. (2019). Investigating the utility of teletherapy in individuals with primary progressive aphasia. Clin. Interv. Aging.

[87] Kiran S., Peña E., Bedore L., Sheng L. Evaluating the Relationship between Category Generation and Language Use and Proficiency. Presented at the Donostia Workshop on Neurobilingualism.

[88] Beeson P.M., Egnor H. (2006). Combining treatment for written and spoken naming. J. Int. Neuropsychol. Soc..

[89] Coltheart M. (1981). The MRC Psycholinguistic Database. Q. J. Exp. Psychol. Sect. A.

[90] Davies M. (2010). The Corpus of Contemporary American English as the first reliable monitor corpus of English. Lit. Linguist. Comput..

[91] Marian V., Bartolotti J., Chabal S., Shook A. (2012). CLEARPOND: Cross-Linguistic Easy-Access Resource for Phonological and Orthographic Neighborhood Densities. PLoS ONE.

[92] Davies M. (2006). Corpus Del Español: Two Billion Words, 21 Countries. http://www.corpusdelespanol.org/web-dial/.

[93] Duchon A., Perea M., Sebastian-Galles N., Martí M.A., Carreiras M. (2013). EsPal: One-stop shopping for Spanish word properties. Behav. Res. Methods.

[94] New B., Pallier C., Brysbaert M., Ferrand L. (2004). Lexique 2: A new French lexical database. Behav. Res. Methods Instrum. Comput..

[95] Davies M. Corpus Do Português: One Billion Words, 4 Countries. http://www.corpusdoportugues.org/web-dial/.

[96] Kilgarriff A., Baisa V., Bušta J., Jakubíček M., Kovář V., Michelfeit J., Rychlý P., Suchomel V. (2014). The Sketch Engine: Ten years on. Lexicography.

[97] Rasooli M.S., Kouhestani M., Moloodi A. Development of a Persian Syntactic Dependency Treebank. Proceedings of the NAACL-HLT.

[98] Dial H., Martin R. (2017). Evaluating the relationship between sublexical and lexical processing in speech perception: Evidence from aphasia. Neuropsychologia.

[99] Kertesz A. (2012). Western Aphasia Battery—Revised.

[100] Kaplan E., Goodglass H., Weintraub S. (2001). Boston Naming Test.

[101] Paul N., Mehrhoff J. (2015). Descriptive Analysis: Survey of Direct and Indirect Interventions for Persons with Dementia-Based Communication Disorders. Perspect. Neurophysiol. Neurogenic Speech Lang. Disord..

[102] Francis W.S. (1999). Cognitive integration of language and memory in bilinguals: Semantic representation. Psychol. Bull..

[103] Francis W.S. (2018). Shared core meanings and shared associations in bilingual semantic memory: Evidence from research on implicit memory. Int. J. Biling..

[104] Hoshino N., Kroll J.F. (2008). Cognate effects in picture naming: Does cross-language activation survive a change of script?. Cognition.

[105] Mendez M.F., Perryman K.M., Pontón M.O., Cummings J.L. (1999). Bilingualism and Dementia. J. Neuropsychiatry Clin. Neurosci..

[106] Ivanova I., Salmon D.P., Gollan T.H. (2014). Which Language Declines More? Longitudinal versus Cross-sectional Decline of Picture Naming in Bilinguals with Alzheimer’s Disease. J. Int. Neuropsychol. Soc..

[107] Henry M.L., Wilson S.M., Babiak M.C., Mandelli M.L., Beeson P.M., Miller Z.A., Gorno-Tempini M.L. (2016). Phonological Processing in Primary Progressive Aphasia. J. Cogn. Neurosci..

[108] Graham K.S., Patterson K., Pratt K.H., Hodges J.R. (2001). Can repeated exposure to “forgotten” vocabulary help alleviate word-finding difficulties in semantic dementia? An illustrative case study. Neuropsychol. Rehabil..

[109] Jokel R., Rochon E., Leonard C. (2006). Treating anomia in semantic dementia: Improvement, maintenance, or both?. Neuropsychol. Rehabil..

[110] Lind M., Simonsen H.G., Ribu I.S.B., Svendsen B.A., Svennevig J., De Bot K. (2017). Lexical access in a bilingual speaker with dementia: Changes over time. Clin. Linguist. Phon..

[111] Ou L., Przybilla M., Koniar B., Whitley C.B. (2018). RTB lectin-mediated delivery of lysosomal α-L-iduronidase mitigates disease manifestations systemically including the central nervous system. Mol. Genet. Metab..

[112] Mahendra N., Spicer J. (2014). Access to Speech-Language Pathology Services for African-American Clients with Aphasia: A Qualitative Study Racial and Ethnic Disparities in Stroke Care. Div. 14 Newsl..

[113] Prince M., Ali G.-C., Guerchet M., Prina M., Albanese E., Wu Y.-T. (2016). Recent global trends in the prevalence and incidence of dementia, and survival with dementia. Alzheimer’s Res. Ther..

[114] Prince M., Acosta D., Albanese E., Arizaga R., Ferri C.P., Guerra M., Huang Y., Jacob K., Jiménez-Velázquez I.Z., Rodriguez J.L. (2008). Ageing and dementia in low and middle income countries–Using research to engage with public and policy makers. Int. Rev. Psychiatry.

[115] Ortman J.M., Shin H.B. Language Projections: 2010 to 2020. Presented at the Annual Meetings of the American Sociological Association.

[116] Zeigler K., Camarota S.A. (2019). 67.3 Million in the United States Spoke a Foreign Language at Home in 2018.

[117] Grandpierre V., Milloy V., Sikora L., Fitzpatrick E., Thomas R., Potter B. (2018). Barriers and facilitators to cultural competence in rehabilitation services: A scoping review. BMC Health Serv. Res..

