# Supplementary material for: Treatment for Anomia in Bilingual Speakers with Progressive Aphasia
Source: Brain Sci. 2021 Oct 20;11(11):1371. doi: 10.3390/brainsci11111371 (PMC8615710; doi:10.3390/brainsci11111371)
Supplement: Supplementary file 1 [file brainsci-11-01371-s001.zip › brainsci-1373951-SI/brainsci-1373951-suppl-update/Supplemental_Mats/Supplemental_mats_part1.pdf]

Supplemental Material: Results of Statistical Analyses at the Single-Subject Level for all Participants

Trained Items

		Pre-Mid				Pre-Post				Pre-3				Pre-6				Pre-12			
ID	Language Dominance Status	p-value	CI Lower Bound	CI Upper Bound	Observed Change	p-value	CI Lower Bound	CI Upper Bound	Observed Change	p-value	CI Lower Bound	CI Upper Bound	Observed Change	p-value	CI Lower Bound	CI Upper Bound	Observed Change	p-value	CI Lower Bound	CI Upper Bound	Observed Change
FTD_RT_1	Dom	**0.00**	58.33	100.00	0.82	**0.00**	41.67	100.00	0.74	**0.00**	50.00	100.00	0.78	**0.00**	66.67	100.00	0.86	**0.00**	41.67	91.67	0.69
SV1	Dom	0.09	-8.33	50.00	0.21	**0.00**	75.00	100.00	0.92	**0.00**	75.00	100.00	0.92	**0.00**	75.00	100.00	0.92	**0.00**	58.33	100.00	0.84
SV2	Dom	**0.00**	66.67	100.00	0.86	**0.00**	66.67	100.00	0.86	**0.00**	50.00	100.00	0.78	**0.00**	66.67	100.00	0.86	**0.00**	50.00	100.00	0.78
SV3	Dom	**0.00**	37.50	79.17	0.59	**0.00**	25.00	66.67	0.46	**0.00**	20.83	62.50	0.42	**0.00**	25.00	66.67	0.46	0.13	-4.17	25.00	0.09
SV4	Dom	**0.00**	83.33	100.00	0.95	**0.00**	79.17	100.00	0.91	NA	NA	NA	NA	NA	NA	NA	NA	NA	NA	NA	NA
LV1	Dom	**0.00**	54.17	91.67	0.75	**0.00**	41.67	83.33	0.63	NA	NA	NA	NA	NA	NA	NA	NA	**0.00**	8.33	54.17	0.32
LV2	Dom	**0.00**	58.33	95.83	0.79	**0.00**	75.00	100.00	0.90	**0.00**	79.17	100.00	0.92	**0.00**	45.83	87.50	0.67	**0.00**	33.33	75.00	0.54
LV3	Dom	**0.00**	75.00	100.00	0.90	**0.00**	12.50	50.00	0.31	**0.00**	8.33	41.67	0.25	0.07	0.00	20.83	0.08	0.19	0.00	12.50	0.04
LV4	Dom	**0.00**	33.33	79.17	0.58	**0.00**	54.17	91.67	0.74	**0.00**	62.50	91.67	0.78	**0.00**	50.00	87.50	0.70	**0.00**	37.50	83.33	0.61
LV5	Dom	0.06	-8.33	58.33	0.25	**0.00**	25.00	83.33	0.57	**0.00**	33.33	91.67	0.67	**0.00**	25.00	83.33	0.59	**0.00**	16.67	83.33	0.50
FTD_RT_1	Nondom	0.18	0.00	25.00	0.08	**0.00**	33.33	91.67	0.63	**0.00**	33.33	83.33	0.58	**0.00**	75.00	100.00	0.92	**0.00**	58.33	100.00	0.83
SV1	Nondom	**0.00**	83.33	100.00	0.97	**0.00**	16.67	75.00	0.47	**0.00**	58.33	100.00	0.80	**0.00**	41.67	91.67	0.72	**0.00**	33.33	91.67	0.64
SV2	Nondom	0.46	-16.67	16.67	0.01	**0.00**	83.33	100.00	0.97	**0.00**	83.33	100.00	0.97	**0.00**	58.33	100.00	0.80	**0.00**	83.33	100.00	0.97
SV3	Nondom	0.81	-12.50	0.00	-0.04	**0.00**	33.33	75.00	0.54	**0.00**	8.33	50.00	0.29	**0.00**	33.33	75.00	0.54	0.81	-12.50	0.00	-0.04
SV4	Nondom	0.50	0.00	0.00	0.00	**0.00**	70.83	95.83	0.85	NA	NA	NA	NA	NA	NA	NA	NA	NA	NA	NA	NA
LV1	Nondom	0.37	-8.33	16.67	0.02	**0.00**	45.83	83.33	0.65	NA	NA	NA	NA	NA	NA	NA	NA	**0.03**	0.00	33.33	0.17
LV2	Nondom	0.44	-8.33	12.50	0.01	**0.00**	12.50	50.00	0.30	**0.00**	33.33	75.00	0.55	**0.00**	8.33	45.83	0.26	0.76	-12.50	0.00	-0.03
LV3	Nondom	0.50	0.00	0.00	0.00	**0.00**	62.50	91.67	0.78	**0.00**	8.33	41.67	0.25	**0.00**	8.33	41.67	0.25	0.50	0.00	0.00	0.00
LV4	Nondom	**0.00**	66.67	95.83	0.83	**0.00**	66.67	95.83	0.83	**0.00**	66.67	95.83	0.83	**0.00**	62.50	95.83	0.79	**0.00**	54.17	91.67	0.75
LV5	Nondom	**0.00**	25.00	91.67	0.60	**0.00**	16.67	75.00	0.48	**0.00**	33.33	91.67	0.61	**0.00**	16.67	83.33	0.53	**0.02**	0.00	66.67	0.36

Untrained Items

		Pre-Mid				Pre-Post				Pre-3				Pre-6				Pre-12			
Participant ID	Language Dominance Status	p-value	CI Lower Bound	CI Upper Bound	Observed Change	p-value	CI Lower Bound	CI Upper Bound	Observed Change	p-value	CI Lower Bound	CI Upper Bound	Observed Change	p-value	CI Lower Bound	CI Upper Bound	Observed Change	p-value	CI Lower Bound	CI Upper Bound	Observed Change
FTD_RT_1	Dom	0.09	-8.33	58.33	0.24	**0.03**	0.00	66.67	0.36	**0.02**	0.00	66.67	0.36	0.13	-16.67	50.00	0.19	0.06	-8.33	58.33	0.28
SV1	Dom	**0.03**	0.00	58.54	0.32	**0.00**	50.00	100.00	0.75	**0.00**	16.67	83.33	0.50	**0.01**	8.33	66.67	0.38	0.30	-16.67	33.33	0.07
SV2	Dom	0.32	-8.33	25.00	0.05	0.65	-16.67	0.00	-0.03	0.65	-16.67	0.00	-0.03	0.65	-16.67	0.00	-0.03	0.65	-16.67	0.00	-0.03
SV3	Dom	0.14	-8.33	29.17	0.11	0.31	-12.50	20.83	0.05	0.93	-20.83	0.00	-0.08	0.17	-8.33	29.17	0.09	0.51	-16.67	16.67	0.00
SV4	Dom	**0.05**	0.00	33.33	0.15	**0.02**	4.17	41.67	0.21	NA	NA	NA	NA	NA	NA	NA	NA	NA	NA	NA	NA
LV1	Dom	**0.00**	12.50	58.33	0.37	**0.00**	16.67	62.50	0.39	NA	NA	NA	NA	NA	NA	NA	NA	0.18	-12.50	29.17	0.10
LV2	Dom	**0.00**	8.33	41.67	0.23	**0.00**	12.50	45.83	0.27	**0.00**	16.67	54.17	0.36	**0.00**	4.17	37.50	0.21	**0.00**	4.17	37.50	0.21
LV3	Dom	**0.04**	0.00	25.00	0.12	**0.00**	20.83	62.50	0.41	**0.00**	8.33	41.67	0.24	0.60	-4.17	0.00	-0.01	0.61	-4.17	0.00	-0.01
LV4	Dom	0.32	-25.00	50.00	0.10	0.40	-37.50	37.50	0.05	0.06	-12.50	75.00	0.35	**0.02**	0.00	87.50	0.48	0.29	-25.00	50.00	0.12
LV5	Dom	0.08	-4.17	41.67	0.17	**0.02**	0.00	50.00	0.26	0.17	-12.50	33.33	0.11	0.17	-12.50	33.33	0.11	0.17	-12.50	33.33	0.11
FTD_RT_1	Nondom	0.18	0.00	25.00	0.08	0.18	0.00	25.00	0.08	0.18	0.00	25.00	0.08	0.18	0.00	25.00	0.08	0.18	0.00	25.00	0.08
SV1	Nondom	0.14	0.00	33.33	0.10	0.06	0.00	41.67	0.17	0.10	0.00	33.33	0.13	**0.03**	0.00	41.67	0.21	0.18	0.00	25.00	0.08
SV2	Nondom	0.50	-16.67	16.67	0.00	0.22	-16.67	33.33	0.10	0.09	-8.33	50.00	0.19	0.21	-8.33	33.33	0.11	0.43	-16.67	25.00	0.02
SV3	Nondom	0.81	-12.50	0.00	-0.04	0.81	-12.50	0.00	-0.04	0.81	-12.50	0.00	-0.04	0.81	-12.50	0.00	-0.04	0.81	-12.50	0.00	-0.04
SV4	Nondom	0.50	0.00	0.00	0.00	**0.02**	0.00	29.17	0.13	NA	NA	NA	NA	NA	NA	NA	NA	NA	NA	NA	NA
LV1	Nondom	**0.00**	12.50	50.00	0.32	**0.00**	12.50	50.00	0.30	NA	NA	NA	NA	NA	NA	NA	NA	0.27	-4.17	12.50	0.03
LV2	Nondom	0.18	-4.17	16.67	0.05	0.28	-4.17	12.50	0.03	0.61	-4.17	0.00	-0.01	0.60	-4.17	0.00	-0.01	0.27	-4.17	12.50	0.03
LV3	Nondom	0.50	0.00	0.00	0.00	0.50	0.00	0.00	0.00	0.50	0.00	0.00	0.00	0.50	0.00	0.00	0.00	0.50	0.00	0.00	0.00
LV4	Nondom	**0.03**	0.00	87.50	0.44	**0.04**	0.00	75.00	0.38	0.27	-25.00	50.00	0.13	**0.01**	12.50	87.50	0.51	**0.05**	0.00	75.00	0.38
LV5	Nondom	**0.04**	-4.17	41.67	0.21	**0.04**	-4.17	41.67	0.21	0.13	-8.33	33.33	0.13	**0.04**	-4.17	41.67	0.21	**0.04**	-4.17	41.67	0.21

Trained Cognates																					
		Pre-Mid				Pre-Post				Pre-3				Pre-6				Pre-12			
Participant ID	Language Dominance Status	p-value	CI Lower Bound	CI Upper Bound	Observed Change	p-value	CI Lower Bound	CI Upper Bound	Observed Change	p-value	CI Lower Bound	CI Upper Bound	Observed Change	p-value	CI Lower Bound	CI Upper Bound	Observed Change	p-value	CI Lower Bound	CI Upper Bound	Observed Change
FTD_RT_1	Dom	**0.02**	0.00	100.00	0.67	**0.00**	33.33	100.00	0.83	**0.00**	100.00	100.00	1.00	**0.00**	100.00	100.00	1.00	**0.02**	0.00	100.00	0.67
SV1	Dom	**0.01**	20.00	100.00	0.67	**0.01**	20.00	100.00	0.67	**0.01**	20.00	100.00	0.67	0.05	0.00	100.00	0.47	**0.01**	20.00	100.00	0.67
SV2	Dom	0.75	-33.33	0.00	-0.11	**0.00**	50.00	100.00	0.81	**0.01**	16.67	100.00	0.56	**0.01**	16.67	100.00	0.56	0.18	-16.67	66.67	0.22
SV3	Dom	0.27	-16.67	33.33	0.09	0.17	-16.67	41.67	0.14	0.13	-8.33	50.00	0.17	0.50	-25.00	25.00	0.00	0.82	-25.00	0.00	-0.08
SV4	Dom	0.21	-16.67	33.33	0.11	**0.01**	8.33	66.67	0.36	NA	NA	NA	NA	NA	NA	NA	NA	NA	NA	NA	NA
LV1	Dom	0.08	-8.33	50.00	0.22	**0.00**	50.00	100.00	0.79	NA	NA	NA	NA	NA	NA	NA	NA	0.14	-8.33	41.87	0.17
LV2	Dom	**0.00**	8.33	66.67	0.38	**0.00**	50.00	100.00	0.75	**0.00**	41.67	91.67	0.67	**0.00**	50.00	100.00	0.75	**0.00**	25.00	75.00	0.50
LV3	Dom	0.50	0.00	0.00	0.00	0.50	0.00	0.00	0.00	0.50	0.00	0.00	0.00	0.18	0.00	25.00	0.08	0.50	0.00	0.00	0.00
LV4	Dom	**0.00**	50.00	100.00	0.78	**0.00**	50.00	100.00	0.78	**0.00**	50.00	100.00	0.78	**0.00**	50.00	100.00	0.78	**0.00**	50.00	100.00	0.78
LV5	Dom	**0.00**	25.00	83.33	0.59	**0.02**	0.00	66.67	0.34	**0.02**	0.00	66.67	0.34	**0.00**	25.00	83.33	0.59	0.14	-8.33	41.87	0.17
FTD_RT_1	Nondom	0.30	-16.67	50.00	0.11	**0.00**	33.33	100.00	0.77	**0.01**	16.67	100.00	0.61	**0.01**	16.67	100.00	0.61	**0.01**	16.67	100.00	0.61
SV1	Nondom	0.50	0.00	0.00	0.00	**0.04**	0.00	66.67	0.33	**0.05**	0.00	66.67	0.33	**0.16**	0.00	50.00	0.17	**0.05**	0.00	66.67	0.33
SV2	Nondom	**0.00**	50.00	100.00	0.83	**0.00**	50.00	100.00	0.83	**0.01**	16.67	83.33	0.50	**0.00**	50.00	100.00	0.83	0.50	0.00	0.00	0.00
SV3	Nondom	0.50	0.00	0.00	0.00	0.50	0.00	0.00	0.00	0.50	0.00	0.00	0.00	0.50	0.00	0.00	0.00	0.50	0.00	0.00	0.00
SV4	Nondom	0.06	0.00	41.67	0.17	0.05	0.00	41.67	0.17	NA	NA	NA	NA	NA	NA	NA	NA	NA	NA	NA	NA
LV1	Nondom	**0.00**	16.67	75.00	0.48	**0.01**	8.33	66.67	0.36	NA	NA	NA	NA	NA	NA	NA	NA	0.23	-16.67	33.33	0.10
LV2	Nondom	**0.00**	25.00	83.33	0.55	**0.00**	16.67	75.00	0.47	**0.02**	0.00	58.33	0.30	**0.00**	16.67	75.00	0.47	0.05	0.00	50.00	0.22
LV3	Nondom	0.50	0.00	0.00	0.00	0.50	0.00	0.00	0.00	0.50	0.00	0.00	0.00	0.50	0.00	0.00	0.00	0.50	0.00	0.00	0.00
LV4	Nondom	0.16	-16.67	66.67	0.26	**0.01**	16.67	100.00	0.59	**0.00**	50.00	100.00	0.84	**0.00**	16.67	100.00	0.67	**0.00**	50.00	100.00	0.84
LV5	Nondom	0.14	-16.67	50.00	0.19	**0.01**	8.33	75.00	0.44	**0.00**	16.67	83.33	0.53	**0.01**	8.33	75.00	0.44	0.25	-16.67	41.67	0.11

Untrained Cognates																					
		Pre-Mid				Pre-Post				Pre-3				Pre-6				Pre-12			
Participant ID	Language Dominance Status	p-value	CI Lower Bound	CI Upper Bound	Observed Change	p-value	CI Lower Bound	CI Upper Bound	Observed Change	p-value	CI Lower Bound	CI Upper Bound	Observed Change	p-value	CI Lower Bound	CI Upper Bound	Observed Change	p-value	CI Lower Bound	CI Upper Bound	Observed Change
FTD_RT_1	Dom	0.17	-16.67	66.67	0.25	0.11	-16.67	83.33	0.33	0.11	-16.67	83.33	0.33	0.50	-33.33	33.33	0.00	0.26	-33.33	66.67	0.17
SV1	Dom	0.06	-16.67	83.33	0.39	**0.00**	33.33	100.00	0.72	**0.03**	0.00	83.33	0.47	0.06	-16.67	83.33	0.39	0.18	-16.67	66.67	0.22
SV2	Dom	0.66	-33.33	0.00	-0.06	0.66	-33.33	0.00	-0.06	0.66	-33.33	0.00	-0.06	0.66	-33.33	0.00	-0.06	0.66	-33.33	0.00	-0.06
SV3	Dom	0.46	-16.67	16.67	0.01	0.20	-8.33	33.33	0.10	0.65	-16.67	0.00	-0.03	0.31	-8.33	25.00	0.05	0.65	-16.67	0.00	-0.03
SV4	Dom	0.05	0.00	50.00	0.24	0.08	-8.33	50.00	0.20	NA	NA	NA	NA	NA	NA	NA	NA	NA	NA	NA	NA
LV1	Dom	**0.00**	16.67	83.33	0.50	**0.01**	8.33	75.00	0.42	NA	NA	NA	NA	NA	NA	NA	NA	0.26	-16.67	33.33	0.09
LV2	Dom	**0.02**	0.00	50.00	0.23	**0.01**	8.33	58.33	0.27	**0.00**	8.33	66.67	0.36	**0.00**	8.33	66.67	0.36	**0.01**	8.33	50.00	0.27
LV3	Dom	0.06	0.00	41.67	0.17	**0.00**	16.67	66.67	0.42	**0.02**	0.00	50.00	0.25	0.50	0.00	0.00	0.00	0.50	0.00	0.00	0.00
LV4	Dom	**0.04**	0.00	100.00	0.58	0.16	-25.00	100.00	0.33	0.16	-25.00	100.00	0.33	**0.04**	0.00	100.00	0.58	0.16	-25.00	100.00	0.33
LV5	Dom	0.26	-16.67	41.67	0.11	0.06	-8.33	58.33	0.28	0.42	-25.00	33.33	0.03	0.25	-16.67	41.67	0.11	0.68	-33.33	16.67	-0.06
FTD_RT_1	Nondom	0.17	0.00	50.00	0.17	0.16	0.00	50.00	0.17	0.17	0.00	50.00	0.17	0.16	0.00	50.00	0.17	0.17	0.00	50.00	0.17
SV1	Nondom	0.17	0.00	50.00	0.17	0.06	0.00	66.67	0.29	0.17	0.00	50.00	0.17	0.09	0.00	66.67	0.25	0.17	0.00	50.00	0.17
SV2	Nondom	0.50	0.00	0.00	0.00	0.09	0.00	66.67	0.25	**0.05**	0.00	66.67	0.33	**0.04**	0.00	66.67	0.33	**0.05**	0.00	66.67	0.33
SV3	Nondom	0.77	-25.00	0.00	-0.06	0.76	-25.00	0.00	-0.06	0.77	-25.00	0.00	-0.06	0.77	-25.00	0.00	-0.06	0.76	-25.00	0.00	-0.06
SV4	Nondom	0.50	0.00	0.00	0.00	**0.01**	0.00	50.00	0.25	NA	NA	NA	NA	NA	NA	NA	NA	NA	NA	NA	NA
LV1	Nondom	**0.00**	16.67	83.33	0.51	**0.00**	25.00	83.33	0.51	NA	NA	NA	NA	NA	NA	NA	NA	0.32	-8.33	25.00	0.05
LV2	Nondom	0.18	-8.33	33.33	0.11	0.29	-8.33	25.00	0.06	0.65	-16.67	0.00	-0.03	0.65	-16.67	0.00	-0.03	0.29	-8.33	25.00	0.06
LV3	Nondom	0.50	0.00	0.00	0.00	0.50	0.00	0.00	0.00	0.50	0.00	0.00	0.00	0.50	0.00	0.00	0.00	0.50	0.00	0.00	0.00
LV4	Nondom	0.26	-50.00	75.00	0.21	0.16	-25.00	100.00	0.33	0.40	-50.00	50.00	0.08	0.76	-50.00	0.00	-0.17	0.40	-50.00	75.00	0.08
LV5	Nondom	0.26	-16.67	41.67	0.11	0.13	-16.67	50.00	0.19	0.25	-16.67	41.67	0.11	0.14	-16.67	50.00	0.19	0.25	-16.67	41.67	0.11

Trained Noncognates

		Pre-Mid				Pre-Post				Pre-3				Pre-6				Pre-12			
Participant ID	Language Dominance Status	p-value	CI Lower Bound	CI Upper Bound	Observed Change	p-value	CI Lower Bound	CI Upper Bound	Observed Change	p-value	CI Lower Bound	CI Upper Bound	Observed Change	p-value	CI Lower Bound	CI Upper Bound	Observed Change	p-value	CI Lower Bound	CI Upper Bound	Observed Change
FTD_RT_1	Dom	0.50	0.00	0.00	0.00	0.50	0.00	0.00	0.00	0.15	0.00	100.00	0.33	0.50	0.00	0.00	0.00	0.50	0.00	0.00	0.00
SV1	Dom	NA	NA	NA	NA	NA	NA	NA	NA	NA	NA	NA	NA	NA	NA	NA	NA	NA	NA	NA	NA
SV2	Dom	0.17	0.00	50.00	0.17	0.09	0.00	66.67	0.25	**0.05**	0.00	66.67	0.33	0.16	0.00	50.00	0.17	0.17	0.00	50.00	0.17
SV3	Dom	0.28	-16.67	33.33	0.08	0.40	-16.67	25.00	0.03	0.73	-16.67	0.00	-0.05	0.39	-16.67	25.00	0.03	0.73	-16.67	0.00	-0.05
SV4	Dom	0.66	-16.67	0.00	-0.03	0.32	-8.33	25.00	0.05	NA	NA	NA	NA	NA	NA	NA	NA	NA	NA	NA	NA
LV1	Dom	**0.00**	16.67	83.33	0.53	**0.00**	25.00	83.33	0.57	NA	NA	NA	NA	NA	NA	NA	NA	0.25	-16.67	41.67	0.11
LV2	Dom	0.07	-8.33	50.00	0.23	**0.01**	8.33	66.67	0.40	**0.00**	16.67	83.33	0.52	**0.00**	16.67	75.00	0.44	0.21	-16.67	33.33	0.11
LV3	Dom	0.31	0.00	16.67	0.04	0.05	0.00	41.67	0.17	0.05	0.00	41.67	0.17	0.50	0.00	0.00	0.00	0.18	0.00	25.00	0.08
LV4	Dom	**0.00**	16.67	100.00	0.58	**0.00**	50.00	100.00	0.83	**0.00**	50.00	100.00	0.83	**0.01**	16.67	83.33	0.50	**0.01**	16.67	83.33	0.50
LV5	Dom	**0.03**	0.00	58.33	0.28	**0.05**	0.00	50.00	0.24	0.40	-16.67	25.00	0.03	**0.03**	0.00	58.33	0.28	0.19	-8.33	33.33	0.12
FTD_RT_1	Nondom	0.50	0.00	0.00	0.00	0.50	0.00	0.00	0.00	0.50	0.00	0.00	0.00	0.16	0.00	50.00	0.17	0.50	0.00	0.00	0.00
SV1	Nondom	0.50	0.00	0.00	0.00	0.50	0.00	0.00	0.00	0.50	0.00	0.00	0.00	0.50	0.00	0.00	0.00	0.50	0.00	0.00	0.00
SV2	Nondom	0.57	-33.33	33.33	-0.03	0.39	-33.33	50.00	0.06	0.40	-33.33	50.00	0.06	0.39	-33.33	50.00	0.06	0.75	-33.33	0.00	-0.11
SV3	Nondom	0.50	0.00	0.00	0.00	0.50	0.00	0.00	0.00	0.50	0.00	0.00	0.00	0.50	0.00	0.00	0.00	0.50	0.00	0.00	0.00
SV4	Nondom	0.50	0.00	0.00	0.00	0.50	0.00	0.00	0.00	NA	NA	NA	NA	NA	NA	NA	NA	NA	NA	NA	NA
LV1	Nondom	0.66	-16.67	0.00	-0.03	0.65	-16.67	0.00	-0.03	NA	NA	NA	NA	NA	NA	NA	NA	0.65	-16.67	0.00	-0.03
LV2	Nondom	0.66	-16.67	0.00	-0.03	0.46	-16.67	16.67	0.01	0.12	-8.33	41.67	0.14	0.13	-8.33	41.67	0.14	0.32	-8.33	25.00	0.05
LV3	Nondom	0.50	0.00	0.00	0.00	0.50	0.00	0.00	0.00	0.50	0.00	0.00	0.00	0.50	0.00	0.00	0.00	0.50	0.00	0.00	0.00
LV4	Nondom	**0.02**	0.00	100.00	0.56	**0.03**	0.00	83.33	0.47	0.06	0.00	83.33	0.39	**0.00**	33.33	100.00	0.72	0.18	-16.67	66.67	0.22
LV5	Nondom	0.10	-8.33	50.00	0.22	**0.04**	0.00	58.33	0.30	0.18	-16.67	41.67	0.14	0.19	-16.67	41.67	0.14	0.60	-25.00	16.67	-0.03

Untrained Noncognates

		Pre-Mid				Pre-Post				Pre-3				Pre-6				Pre-12			
Participant ID	Language Dominance Status	p-value	CI Lower Bound	CI Upper Bound	Observed Change	p-value	CI Lower Bound	CI Upper Bound	Observed Change	p-value	CI Lower Bound	CI Upper Bound	Observed Change	p-value	CI Lower Bound	CI Upper Bound	Observed Change	p-value	CI Lower Bound	CI Upper Bound	Observed Change
FTD_RT_1	Dom	0.18	-16.67	66.67	0.22	0.11	-16.67	83.33	0.31	**0.00**	33.33	100.00	0.72	0.06	-16.67	83.33	0.39	0.06	0.00	83.33	0.39
SV1	Dom	0.11	-16.67	66.67	0.28	0.06	0.00	83.33	0.36	0.06	0.00	83.33	0.36	0.29	-16.67	50.00	0.11	0.65	-33.33	0.00	-0.06
SV2	Dom	0.17	0.00	50.00	0.17	0.16	0.00	50.00	0.17	0.50	0.00	0.00	0.00	0.50	0.00	0.00	0.00	0.50	0.00	0.00	0.00
SV3	Dom	0.14	-16.67	50.00	0.19	0.13	-16.67	50.00	0.19	0.25	-16.67	41.67	0.11	0.25	-16.67	41.67	0.11	0.42	-25.00	33.33	0.03
SV4	Dom	0.27	-8.33	25.00	0.06	0.41	-8.33	16.67	0.02	NA	NA	NA	NA	NA	NA	NA	NA	NA	NA	NA	NA
LV1	Dom	0.09	-8.33	58.33	0.24	**0.03**	0.00	66.67	0.36	NA	NA	NA	NA	NA	NA	NA	NA	0.25	-16.67	41.67	0.11
LV2	Dom	**0.02**	0.00	50.00	0.25	**0.01**	8.33	58.33	0.29	0.05	0.00	41.67	0.17	0.18	0.00	25.00	0.08	0.05	0.00	41.67	0.17
LV3	Dom	0.32	-8.33	25.00	0.05	0.13	-8.33	41.67	0.14	0.65	-16.67	0.00	-0.03	0.65	-16.67	0.00	-0.03	0.65	-16.67	0.00	-0.03
LV4	Dom	0.17	-25.00	100.00	0.33	**0.00**	50.00	100.00	0.83	0.16	-25.00	100.00	0.33	0.16	-25.00	100.00	0.33	**0.04**	0.00	100.00	0.58
LV5	Dom	0.06	-8.33	58.33	0.28	0.09	-8.33	58.33	0.24	0.13	-16.67	50.00	0.19	0.25	-16.67	41.67	0.11	0.06	-8.33	58.33	0.28
FTD_RT_1	Nondom	0.50	0.00	0.00	0.00	0.50	0.00	0.00	0.00	0.50	0.00	0.00	0.00	0.50	0.00	0.00	0.00	0.50	0.00	0.00	0.00
SV1	Nondom	0.39	0.00	16.67	0.04	0.39	0.00	16.67	0.04	0.30	0.00	33.33	0.08	0.17	0.00	50.00	0.17	0.50	0.00	0.00	0.00
SV2	Nondom	0.57	-33.33	33.33	-0.03	0.75	-33.33	0.00	-0.11	0.76	-33.33	0.00	-0.11	0.39	-33.33	50.00	0.06	0.75	-33.33	0.00	-0.11
SV3	Nondom	0.66	-16.67	0.00	-0.03	0.65	-16.67	0.00	-0.03	0.65	-16.67	0.00	-0.03	0.65	-16.67	0.00	-0.03	0.65	-16.67	0.00	-0.03
SV4	Nondom	0.50	0.00	0.00	0.00	0.50	0.00	0.00	0.00	NA	NA	NA	NA	NA	NA	NA	NA	NA	NA	NA	NA
LV1	Nondom	0.10	0.00	33.33	0.13	0.18	0.00	25.00	0.08	NA	NA	NA	NA	NA	NA	NA	NA	0.50	0.00	0.00	0.00
LV2	Nondom	0.50	0.00	0.00	0.00	0.50	0.00	0.00	0.00	0.50	0.00	0.00	0.00	0.50	0.00	0.00	0.00	0.50	0.00	0.00	0.00
LV3	Nondom	0.50	0.00	0.00	0.00	0.50	0.00	0.00	0.00	0.50	0.00	0.00	0.00	0.50	0.00	0.00	0.00	0.50	0.00	0.00	0.00
LV4	Nondom	**0.02**	0.00	100.00	0.67	**0.00**	25.00	100.00	0.79	**0.01**	25.00	100.00	0.67	**0.00**	50.00	100.00	0.92	**0.01**	25.00	100.00	0.67
LV5	Nondom	0.06	-8.33	58.33	0.27	**0.03**	0.00	66.67	0.34	**0.04**	0.00	66.67	0.31	0.09	-8.33	50.00	0.22	**0.04**	0.00	66.67	0.31

*Note. Significant values shown in bold font.
