# Supplementary material for: Treatment for Anomia in Bilingual Speakers with Progressive Aphasia
Source: Brain Sci. 2021 Oct 20;11(11):1371. doi: 10.3390/brainsci11111371 (PMC8615710; doi:10.3390/brainsci11111371)
Supplement: Supplementary file 1 [file brainsci-11-01371-s001.zip › brainsci-1373951-SI/brainsci-1373951-suppl-update/Supplemental_Mats/Supplemental_mats_part2.pdf]

Supplemental Material. Results Following the Initial Phase of Treatment and Cross-Linguistic Generalization Effects to Untrained Items

1. *Results Following the Initial Phase of Treatment*

Treatment Effects

For pragmatic reasons, all but three participants (sd2, lv4 and lv5) received treatment in their dominant language in the first treatment phase. Therefore, at the mid-treatment timepoint we could examine direct treatment effects in the dominant language for seven participants, and in the nondominant language for the remaining three participants. The seven individuals who received treatment in their dominant language in the first treatment phase demonstrated a significant within-language response at the mid-treatment timepoint (*M* change = 80.81%), and the same was true for the three individuals who received treatment in their nondominant language in the initial phase (*M* change = 80.10%; see Figure 3 and table in supplemental material).

Cross-Linguistic Translation Effects

As previously mentioned, all but three participants (sd2, lv4 and lv5) received treatment in their dominant language in the first phase. Therefore, at the mid-treatment timepoint we could examine translation effects from the dominant to nondominant language for seven participants, and for three individuals from the nondominant to the dominant language. Less than half of the participants (three out of seven) demonstrated a significant transfer effect for cognates from the dominant to nondominant language (*M* change = 30.57%); however, none of the seven participants showed a significant effect for noncognates (*M* change = -1.29%; see Figure 4 and supplemental material). All

participants who received treatment in their nondominant language first showed a significant translation effect for both cognates (n= 3; *M* change = 68.00%) and noncognates (n= 2; *M* change = 43.00%).

2. Cross-Linguistic Generalization Effects to Untrained Items

From pre- to post-treatment four of ten participants demonstrated a significant generalization effect for untrained cognates from pre- to post-treatment in the dominant language (*M* change = 30.11%), and two of ten individuals showed significant transfer in the nondominant language (*M* change = 19.92%; see Figure 4). Of the eight participants who were available for 3-month follow-up, three demonstrated a significant generalization effect for untrained cognates in the dominant language (*M* change = 21.07%) with one individual showing this pattern in the nondominant language (*M* change = 9.58%). At the 6-month follow-up, two individuals (of 8) demonstrated a significant generalization effect for untrained cognates relative to pre-treatment performance in the dominant language (*M* change = 17.86%), and one individual showed generalization in the nondominant language (*M* change = 8.50%). At 12 months post-treatment this pattern persisted for only one individual, in each dominance category (in dominant language *M* change = 10.32%; in nondominant language *M* change = 10.07%).

At post-treatment three of ten individuals showed a significant generalization effect in their dominant language (*M* change = 29.07%) and two of nine individuals showed this pattern in the nondominant language (*M* change = 11.12%). The pattern of performance at follow-ups was similar, with decline noted over time in the number of

participants who showed a significant effect (3 months = one of eight in the dominant, *M* change = 23.17%, two of eight in the nondominant, *M* change = 11.54%; 6 months = zero of eight in the dominant, *M* change = 13.75%, and one of eight to the nondominant, *M* change = 16.71%; 12 months = one of nine to the dominant, *M* change = 16.37% and two of nine to the nondominant, *M* change = 9.33%).

A direct comparison of the magnitude of generalization for untrained cognate and noncognate items revealed a significant difference (with greater cognate transfer) for three participants showed a significant difference in their nondominant language only at post-treatment (see Figure 5 and supplemental material; *M* difference = 8.80%). At follow-up visits, decline in the number of participants who showed this pattern and in the magnitude of this difference was observed (3 months = one of eight in the dominant, *M* difference = -2.10%, one of eight in the nondominant *M* difference = -1.96%; 6 months = one of eight in the dominant, *M* difference = 4.11% and zero of eight in the nondominant, *M* difference = -8.21%; 12 months = zero of nine to the dominant, *M* difference = -6.05%, and one of nine in the nondominant (*M* difference = 0.74%).
